# A multilevel hierarchical framework for quantification of experimental heterogeneity in population snapshot data

**DOI:** 10.1371/journal.pcbi.1014379

**Published:** 2026-06-15

**Authors:** David J. Warne, Xiangrun Zhu, Thomas P. Steele, Stuart T. Johnston, Scott A. Sisson, Matthew Faria, Ryan J. Murphy, Alexander P. Browning

**Affiliations:** 1 School of Mathematical Sciences, Queensland University of Technology, Brisbane, Australia; 2 School of Mathematics and Statistics, The University of Melbourne, Melbourne, Australia; 3 School of Mathematics and Statistics, University of New South Wales, Sydney, Australia; 4 Department of Biomedical Engineering, The University of Melbourne, Melbourne, Australia; 5 School of Mathematical Sciences, Adelaide University, Adelaide, Australia; 6 Mathematical Institute, University of Oxford, Oxford, United Kingdom; Duke University School of Medicine, UNITED STATES OF AMERICA

## Abstract

Biological systems exhibit substantial heterogeneity: that is, variation in specific characteristics of individuals within a population. As a result, it is of critical importance to appropriately account for biological heterogeneity when calibrating mathematical models to infer cellular processes and predict behaviour. Recent approaches consider ordinary differential equations with random parameters to quantify heterogeneity in dynamical processes of cells. In this setting, statistical inference is performed to characterise the distribution of these random parameters within a cell population. One significant limitation of this approach is the tacit assumption that there are no substantial deviations in these distributions across experimental replicates. In this work, we propose a flexible Bayesian hierarchical differential equation modelling framework that quantifies and distinguishes both inter-experimental heterogeneity (heterogeneity between experimental replicates) and intra-experimental heterogeneity (biological heterogeneity within replicate populations). We consider two recent studies that employ mathematical models to interpret flow cytometry snapshot data and quantify heterogeneity in nano-particle cell interactions and cell internalisation processes. Using simulation data, we demonstrate that substantial inaccuracy in the inferred dynamics can arise when experimental heterogeneity is not accounted for. By contrast, our hierarchical approach is robust to variability in inter-experimental and intra-experimental heterogeneity and our method simplifies to previous methods when inter-experimental heterogeneity is negligible. Our approach is flexible and widely applicable to applications involving replicate populations and snapshot data. We provide open-source implementations of our methods on GitHub.

## Author summary

In the design of personalised and targeted drugs and therapeutics it is essential to quantify the variability in the response of cellular processes involved in drug uptake. In practice, the experimental instruments and protocols used to collect cell population data for the study of such cellular processes are complex. As a result, it is important for statistical analyses that use these data to correctly account for variability between experiments in addition to the variability of cellular behaviour within the same experimental replicate. We present a flexible model of cellular dynamics that accounts for variability in biological, technical, and experimental replicates. We show that this leads to more accurate and robust results over standard approaches that pool replicates together.

## Introduction

Accounting for biological heterogeneity is crucial in the biomedical sciences [1]. Heterogeneity plays a key role in the development and maintenance of healthy living organisms [2,3] through complex interactions between various types of cells driven by intracellular biochemical processes [4]. In pathological situations, such as cancer or viral infections [5–8], heterogeneity can seriously impact the efficacy of pharmaceutical interventions and eventual patient outcomes [9–11]. As a result, appropriately quantifying biological heterogeneity within a population of cells is of tremendous importance for understanding natural processes and for the design of effective drugs, especially in the areas of precision medicine such as targeted therapeutics [12–15].

**Fig 1:**
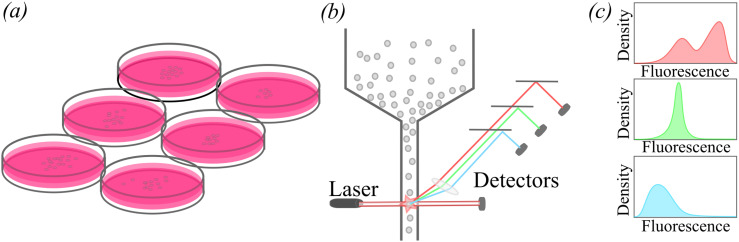
Schematic of flow cytometry data acquisition. **(a)** Cells are seeded into cell culture plates, e.g., 12-well plates, in a growth media (e.g., 1 mL) and incubated with various treatments for some time period (typically between 1 to 48 hours). **(b)** Cells are then removed from plates and placed in suspension, and passed through the flow cytometer nozzle to a laser beam where light scattering from the cell is measured at detectors. **(c)** For each fluorescent channel, the intensity distribution is obtained, leading to distributions showing cellular variability.

A standard experimental approach to quantify properties of cell populations is flow cytometry [16–18]. In such an experiment, populations of cells (Fig 1(a)), are tagged or stained with fluorescent markers. The cells then individually flow through the cytometer nozzle and pass through a laser from which scattered light is amplified and converted to a digital signal representing a measurement of fluorescent intensity related to each cell in the population (Fig 1(b)). This results in a snapshot in time of the fluorescent intensity distribution across multiple colour channels, providing a rich source of information about potential variability in the cell populations at a single point in time (Fig 1(c)). Typically, thousands to tens of thousands of cells are analysed per time point.

Data obtained from flow cytometry experiments exhibit substantial levels of heterogeneity. While some of this variation can be attributed to measurement noise, it is well established that some of this variation arises due to intrinsic biological variation, even between genetically identical cells [19–23]. However, there are also extrinsic factors that drive heterogeneity in flow cytometry experiments that can adversely affect the statistical analysis of the biological heterogeneity of interest if not properly accounted for [24–26]. For example, subtle variation in sample preparation, instrument configuration for data acquisition, and manual user operation can have a substantial effect on the signal-to-noise ratio and statistical bias in the data, potentially impacting the validity of conclusions drawn from the analysis [24,25,27,28].

In many studies, it is typically assumed that experimental replicates of identically prepared cell populations represent independent and identically distributed replicates. For example, in the work of Faria et al. [29], human leukaemia cells (THP-1 cell line) are seeded into a 12-well plate and nano-particle-cell interactions are observed via flow cytometry at six observation times (between 1 hr and 24 hrs), resulting in two replicates per observation time. In practice, statistical analysis is performed on the pooled sample of replicates [30,31], or each replicate is individually analysed [32,33]. Neither approach is ideal. Pooling ignores heterogeneity between experimental replicates and may lead to incorrect conclusions. Conversely, analysing replicates independently can lead to difficulties in assessing global trends [34]. A potential solution to analyse flow cytometry data is to use mathematical or statistical models that structure relationships between snapshots, and between time points data [34–38].

In this work, we introduce an approximate Bayesian multilevel hierarchical modelling framework for quantifying biological heterogeneity in flow cytometry data in situations where the parameters of interest are related to a mathematical model of cell dynamics based on differential equation models. The class of models we consider here is more general than previous work on non-linear mixed-effects models [35,36], as we allow for all cells to interact with a shared dynamic environment. To demonstrate our approach, we consider simulation studies based on two recent studies that quantify biological heterogeneity in cell behaviour using pooled flow cytometry data [39,40]. We highlight the dramatic impact that variation between replicates can have on pooled sample analysis results and show how a hierarchical approach can account for this effect. In addition, our approach reduces to the pooled case in situations where variation between replicates is minimal. Our approach is therefore generally applicable for applications in which controlling variation across flow cytometry replicates is infeasible.

## Materials and methods

**Fig 2:**
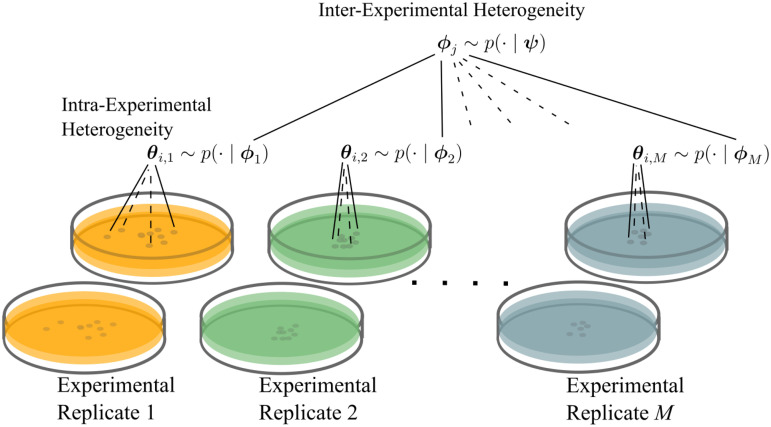
A schematic representation of the hierarchical statistical framework. Experimental replicates are indicated by different colours, with each experiment consisting of two identical technical replicates. Inter-experimental heterogeneity is captured through the hyper-parameters ψ that characterise the distribution of the *M* replicate specific hyper-parameters, $\phi_{1},\phi_{2},\ldots ,\phi_{M}$. Here, $\phi_{j}$ characterises the intra-experimental heterogeneity between $N_{j}$ cells with $\theta_{i,j}$ being the dynamic parameters for the *i*th cell within the *j*th replicate.

We develop a general Bayesian hierarchical modelling framework for the analysis of heterogeneity in cell behaviour using flow cytometry data. This heterogeneity is variability that can be intra-experimental heterogeneity within a replicate, or inter-experimental heterogeneity between replicates. This inter-experimental heterogeneity can be variation between either technical replicates or experimental replicates. For clarity, we define a technical replicate to be an identically prepared replicate performed at the same time and the same person, and define an experimental replicate to be a replicate replicate performed on a different day, and/or by a different person (See Fig 2). For example, if an experiment was performed with a 12-well plate where two-wells were assigned to each of six time points then this would represent two technical replicates. However, if the entire 12-well plate experiment was performed again at a different lab or with a different instrument then this would be two experimental replicates, each with two technical replicates. For most experimental protocols, we expect technical replicates to be closely related. As a result, we focus our attention to experimental replicates in this manuscript. However, we show how this can be extended to account for both technical and experimental replicates in S1 Appendix.

### Flow cytometry data

We use simulated flow cytometry data that is a realistic characterisation of real flow cytometry data. Specifically, we build our data simulation process based on recent studies that investigate heterogeneity in interactions between cells and nano-particles [29,30,40] and cell internalisation processes [39,41,42].

#### Nano-engineered particle-cell interaction data.

The flow cytometry data published by Faria et al., [29] and subsequently analysed for heterogeneity in particle-cell interaction by Murphy et al., [40] are for a human leukaemia monocytic suspension cell line, THP-1. Here, THP-1 cells are seeded in a 12-well plate and incubated with nano-particles (specifically, we focus on the 150 nm core-shell data) for 1, 2, 4, 8, 16, and 24 [hr]. After incubation, nano-particles that are not associated with cells are removed via washing. The nano-particle fluorescence across the cell population from each well plate is collected via flow cytometry. This results in snapshot data of associated nano-particle fluorescence for N≈ 20,000 cells for each incubation time point.

#### Dual-labelled probe internalisation data.

Browning et al., [39] published flow cytometry data for a human B cell lymphoblast cell line (C1R) and analysed the heterogeneity in endocytosis of anti-transferrin (anti-TFR) antibodies. The measurement technique is based upon programmable sensors using DNA quenching probes [43,44]. Antibodies are dual-labelled with fluorescent dyes: FIP-Cy5 and BODIPY FL. Of these two probes, FIP-Cy5, is quenchable, that is, the fluorescence is effectively disabled on the cell surface using a quencher dye. Here, C1R cells are incubated with dual-labelled antibodies with incubation times of 5, 10, 20, 30, 60, 120, and 180 [min]. After incubation, cells are washed and resuspended both with and without the quencher [44]. The fluorescent signals for both FIP-Cy5 and and BODIPY FL are measured for the cell populations in both quenched and unquenched samples via flow cytometry. This leads to snapshots of the jointly distributed FIP-Cy5 and BODIPY FL fluorescence for *N* = 1,000 cells over the incubation times and qenched/unquenched samples.

### Mathematical modelling

We consider applications for which flow cytometry data is analysed using deterministic modelling based on ordinary differential equations (ODEs) as is commonplace in the mathematical and computational biology communities. The goal of such analysis is to quantify biological heterogeneity in parameters related to the dynamics of some cellular process. In this sense, each individual cell in the population is treated as having its own set of parameters and the goal is to find a suitable distribution for the parameters leading to cell dynamics matching the data distribution over time [39,40,45]. Here we describe the general ODE framework with random parameters used in the setting of pooled replicates, and then connect the framework to the particle-cell interaction model of Murphy et al., [40] and the cell internalisation model of Browning et al., [39]. These previous works focus exclusively on intra-experimental heterogeneity. We then show how to extend the analysis framework also account for inter-experimental heterogeneity between replicates and present a Bayesian multilevel hierarchical approach to quantify both intra- and inter-experimental heterogeneity together.

#### Cell population models with random parameters.

We consider a representative and heterogeneous population of *N* cells. Let ${𝐗}_{i}$ be a vector of $d_{X}\geq 1$ dynamic properties of interest related to the *i*th cell (e.g., the average number of nano-particles associated with cell, or concentrations of various biochemicals). This property vector evolves over time according to


$$\begin{array}{cc} \frac{\text{d}{𝐗}_{i}}{\text{d}t} & =f({𝐗}_{i}(t),𝐙(t),t;\theta_{i}),\\ \frac{\text{d}𝐙}{\text{d}t} & =h(𝒳(t),𝐙(t),t;\Theta_{N}), \end{array}$$ (1)

for i=1,2,…,N, where $\theta_{i}$ is a vector of $d_{\theta }\geq 1$ parameters with values specific to the *i*th cell, $\Theta_{N}=[\theta_{1},\theta_{2},\ldots ,\theta_{N}]$ is a $d_{\theta }\times N$ matrix of the population’s parameters, $𝒳(t)=[{𝐗}_{1}(t),{𝐗}_{2}(t),\ldots ,{𝐗}_{N}(t)]$ is a $d_{X}\times N$ matrix representing the state of the cell population, and 𝐙(t) is a vector of $d_{Z}\geq 1$ shared environmental variables that interact with cells in the population (e.g., a shared nutrient source). The functions $f({𝐗}_{i}(t),𝐙(t),t;\theta_{i})$ and $h(𝒳(t),𝐙(t),t;\Theta_{N})$ define the dynamics of cells and the environment, respectively. In this setting, cells only indirectly interact via the shared environment. Direct interaction between cells could be incorporated in our framework by extending the cell dynamics to be a function of 𝒳(t) instead of ${𝐗}_{i}(t)$ only. For *t*_0_ representing the start of the initial incubation time, we have initial conditions ${𝐗}_{i}(t_{0})={𝐱}_{i,0}$ for i=1,2,…,N, and $𝐙(t_{0})={𝐳}_{0}$. Importantly, we do not assume that these initial conditions are known quantities, since such assumptions are known to cause problems in study reproducibility [46]. Instead, initial conditions are included as model parameters that we will estimate. This estimation need not be independent of the other parameters, but rather, initial conditions could be functionally dependent on the model parameters. For example, one may assume the environment or cell states to be in equilibrium that is determined based on a function of rate parameters.

The measured fluorescence of each cell is subject to external noise and thus treated as a random variable, that is,


$$\begin{array}{c} {𝐘}_{i}(t)\sim g(\cdot |{𝐗}_{i}(t),\theta_{i},\eta ), \end{array}$$ (2)

where ${𝐘}_{i}(t)$ are the recorded fluorescent intensities for the *i*th cell given the cell state ${𝐗}_{i}(t)$, dynamics parameters $\theta_{i}$, and observation process specific parameters, η. The functional from of the observation process, $g(\cdot |{𝐗}_{i}(t),\theta_{i},\eta )$, will typically be determined based on calibration data for flow cytometry. The dimension of ${𝐘}_{i}(t)$ will be the number of fluorescence channels and will generally not be the same as the dimension of ${𝐗}_{i}(t)$. Thus a single cell population snapshot obtained at time *t* > *t*_0_ using flow cytometry is given by $𝒴(t)=[{𝐘}_{1}(t),{𝐘}_{2}(t),\ldots ,{𝐘}_{N}(t)]$. Typically, we do not observe the initial condition at *t* = *t*_0_, and therefor it is treated as a latent variable to infer with the model parameters. Finally, for *n* observation times, $t_{1}<t_{2}<\cdots <t_{n}$, we represent the entire flow cytometry da*t*aset as $𝒟=[𝒴(t_{1}),𝒴(t_{2}),\ldots ,𝒴(t_{n})]$.

Since snapshot data as obtained through flow cytometry does not track individual cells, using such data to perform parameter inference on $\theta_{1},\theta_{2},\ldots ,\theta_{N}$ is not meaningful. Instead, one can consider $\theta_{1},\theta_{2},\ldots ,\theta_{N}$ to be independent identically distributed (i.i.d.) samples from a parametric distribution with probability density, p(θ∣ϕ), then perform parameter inference on the hyper-parameters ϕ∈Φ, where Φ is the hyper-parameter space. In a Bayesian setting, this leads to


$$\begin{array}{c} p(\phi |𝒟)\propto p(𝒟|\phi )p(\phi ), \end{array}$$

where


$$\begin{array}{c} p(𝒟|\phi )=\int_{\Theta^{N}}p(𝒟|\theta_{1},\theta_{2},\ldots ,\theta_{N})\left[\prod_{i=1}^{N}p(\theta_{i}|\phi )\;\text{d}\theta_{i}\right]. \end{array}$$

This is the essence of the approach taken by Browning et al., [39] and Murphy et al., [40] and it relies on pooled snapshot data that requires the assumption of i.i.d. parameters across all cells and replicates. Our main contribution, that we present later, is an extension to this framework that only requires i.i.d. parameters within each experimental replicate.

#### Example 1: Particle-cell interaction model.

The particle-cell interaction model of Murphy et al., [40], describes the association of free nano-particles to a population of cells. Given *N* cells the number of particles associated with the *i*th cell at time *t* > *t*_0_, $P_{i}(t)$, is governed by *t*he system,


$$\begin{array}{cc} \frac{\text{d}P_{i}}{\text{d}t} & =r_{i}cs\left(1-\frac{P_{i}(t)}{K_{i}}\right)u(t),\\ \frac{\text{d}u}{\text{d}t} & =-\frac{1}{N}\sum_{i=1}^{N}\frac{r_{i}cs}{v}\left(1-\frac{P_{i}(t)}{K_{i}}\right)u(t), \end{array}$$ (3)

for i=1,2,…,N, where $r_{i}$ and $K_{i}$ are, respectively, the association rate and carrying capacity of particles for the *i*th cell, *c* is the fractional cell surface coverage, *s* is the surface area of the cell boundary, *v* is the volume of the well-mixed media, and *u*(*t*) is the total free particle density with initial condition $u(t_{0})=u_{0}$. In the context of our framework given by Eqs. (1) and (2), ${𝐗}_{i}(t)=P_{i}(t)$ and ${𝐙}_{i}(t)=u(t)$ (i.e., $d_{X}=d_{Z}=1$), and $\theta_{i}=[r_{i},K_{i}{]}^{⊤}$ (i.e., $d_{\theta }=2$).

The measured fluorescence, $Y_{i}(t)$, for cell *i* is modelled by


$$\begin{array}{c} Y_{i}(t)=Y_{\text{cell},i}+\left(P_{i}(t)-\lfloor P_{i}(t)\rfloor \right)Y_{\text{particle},\lceil P_{i}(t)\rceil }+\sum_{j=1}^{\lfloor P_{i}(t)\rfloor }Y_{\text{particle},j}, \end{array}$$

where $Y_{\text{cell},i}\sim p(\cdot |\eta )$ is the autofluorescence of a cell, drawn at random from the empirical distribution cell-only calibration dataset, and $Y_{\text{particle},1},\ldots ,Y_{\text{particle},\lceil P_{i}(t)\rceil }\sim p_{\text{particle}}(\cdot |\eta )$ are individual particle fluorescences drawn at random from the empirical distribution particle-only calibration dataset. Here η are parameters associated with normalising voltages that are used to obtain flow cytometry measurements (See Murphy et al., [40] for details). Since there are *N* = 20,000 cells, this leads to a computationally challenging system of ODEs as they are fully coupled through the environment (Eq. (3)). However, a tractable approximation with a semi-closed form solution can be obtained under the assumption that $u(t)\approx u_{0}$ throughout the simulation time (See Murphy et al., [40] for details). This assumption is quite common in the field and represents a high nano-particle dose such that the cells are not expected to meaningfully deplete it. Importantly, it does not lead to a completely decoupled system free from any environmental coupling and changes in *u*(*t*) are still captured.

#### Example 2: Internalisation model.

Browning et al., [39] consider a model describing the internalisation of transferrin anti-bodies that accounts for receptor recycling. Due to the experimental protocol (See Dual-labelled probe internalisation data), the concentration of free transferrin antibodies can be considered sufficiently high that unbound surface receptors are assumed to bind instantaneously to a free antibody. Furthermore, the high free antibody concentration enables the internalisation dynamics of all *N* = 2,000 cells to be considered independent of each other since cells will never compete for free antibodies. For the *i*th cell at time *t* > *t*_0_, Browning e*t* al., [39] model the dynamics of the density of unbound internal receptors, $T_{i}(t)$, bound surface receptors, $S_{i}(t)$, internalised bound receptors, $E_{i}(t)$, and interalised free antibodies, $F_{i}(t)$, according to,


$$\begin{array}{cc} \frac{\text{d}T_{i}}{\text{d}t} & =-\beta_{i}T_{i}(t),\\ \frac{\text{d}S_{i}}{\text{d}t} & =\beta_{i}T_{i}(t)+p\beta E_{i}(t)-\lambda_{i}S_{i}(t),\\ \frac{\text{d}E_{i}}{\text{d}t} & =\lambda_{i}S_{i}(t)-p\beta_{i}E_{i}(t),\\ \frac{\text{d}F_{i}}{\text{d}t} & =-p\beta_{i}E_{i}(t), \end{array}$$ (4)

for i=1,2…,N. Here, the initial total density of receptors for the *i*th cell is given by $T_{i}(t_{0})+S_{i}(t_{0})=R_{i}$ and it is assumed no antibodies have initially been internalised with $E_{i}(t_{0})=F_{i}(t_{0})=0$. Further, $\lambda_{i}>0$ and $\beta_{i}>0$ are, respectively, the internalisation rate and recycling rate for the the *i*th cell. Finally, p∈(0,1] is the disassociation probability that captures the probability that an internalised antibody is absorbed into the cell versus being recycled back to the surface. Following Browning et al., [39], *p* is associated with a purely chemical process and considered constant across cells, whereas $\lambda_{i}$, $\eta_{i}$ and $R_{i}$ are assumed to be heterogeneous across cells i=1,2,…N. Relating this to our framework (Eqs. (1) and (2)), ${𝐗}_{i}(t)=[T_{i}(t),S_{i}(t),E_{i}(t),F_{i}(t){]}^{⊤}$ (i.e., $d_{X}=4$), and $\theta_{i}=[\lambda_{i},\beta_{i},R_{i},p{]}^{⊤}$ (i.e., $d_{\theta }=4$). In this model we do not have a dependent shared environment so $d_{Z}=0$. In this case an analytic solution to Eq. (4) can be obtained using the matrix exponential (See Browning et al., [39] for details).

The observation process is more complex in this model than the particle-cell interaction model. Here, the experimental protocol involves dual-labelled antibodies with one of the labels (FIP-Cy5) being “quenchable”, leading to a loss in FIP-Cy5 fluorescence for free or surface bound antibodies, and the other (BODIPY FL) being “unquenchable” with fluorescence unaffected by the quenching process. The *N* = 2,000 cells consist of two equal-sized technical replicates of size $\tilde{N}=$ 1,000 with one replicate undergoing the *quenching* process before being processed through the flow cytometer. This leads to four measured fluorescence signal values for the *i*th pair of cells,


$$\begin{array}{c} {𝐘}_{i}(t)=[Q_{i}(t),U_{i}(t),{\bar{Q}}_{i}(t),{\bar{U}}_{i}(t)], \end{array}$$

where $Q_{i}(t)$ (resp. $U_{i}(t)$) are the quenchable FIP-Cy5 (resp. unquenchable BODIPY FL) fluorescence signals measured from the unquenched sample group, and ${\bar{Q}}_{i}(t)$ (resp. ${\bar{U}}_{i}(t)$) are the quenchable FIP-Cy5 (resp. unquenchable BODIPY FL) fluorescence signals measured from the quenched sample group. The resulting observation process is


$$\begin{array}{cc} Q_{i}(t) & \sim 𝒩(\alpha_{1}\left[T_{i}(t)+S_{i}(i)+E_{i}(t)+F_{i}(t)\right]+A_{Q},\sigma_{1}),\\ U_{i}(t) & \sim 𝒩(\alpha_{2}\left[T_{i}(t)+S_{i}(i)+E_{i}(t)+F_{i}(t)\right]+A_{U},\sigma_{2}),\\ {\bar{Q}}_{i}(t) & \sim 𝒩(\alpha_{1}\left[(1-q){\bar{S}}_{i}(i)+{\bar{E}}_{i}(t)+{\bar{F}}_{i}(t)\right]+A_{Q},\sigma_{1}),\\ {\bar{U}}_{i}(t) & \sim 𝒩(\alpha_{2}\left[{\bar{T}}_{i}(t)+{\bar{S}}_{i}(i)+{\bar{E}}_{i}(t)+{\bar{F}}_{i}(t)\right]+A_{U},\sigma_{2}), \end{array}$$ (5)

for $i=1,2,\ldots ,\tilde{N}$, where $\alpha_{1}$ and $\sigma_{1}$ (resp. $\alpha_{2}$ and $\sigma_{2}$) are intensity and noise the fluorescence measurement for FIP-Cy5 (resp. BODIPY FL). Further, $A_{Q}$ (resp. $A_{U}$) corresponds to the average autofluorescence of FIP-Cy5 (resp. BODIPY FL), and *q* is the quenching efficiency. While $A_{Q}$, $A_{U}$ and *q* are pre-estimated from data directly, the parameters $\eta =(\alpha_{1},\alpha_{2},\sigma_{1},\sigma_{2})$ must be inferred with the model parameters.

### Statistical framework

In the previous sections, we describe the typical setting in which biological heterogeneity has been treated in the literature. That is, replicates are pooled and treated as a single snapshot population [29–31,39,40]. In this work, we propose an extension in which each replicate is treated as its own population with variation within replicates (i.e., intra-experimental heterogeneity) being distinguished from variation between replicates (i.e., inter-experimental heterogeneity). To simplify the exposition of our approach we present a two-level Bayesian hierarchical model, however, the approach can be extended to more levels as shown in the S1 Appendix.

#### Inter-experimental and intra-experimental heterogeneity.

Within the random ODE framework (Eqs. (1) and (2)), we consider *M* experimental replicates with the *j*th replicate analysing $N_{j}$ cells in total across identical technical replicates (Fig 2). The cell population dynamic model (Eq. (1)) becomes,


$$\begin{array}{cc} \frac{\text{d}{𝐗}_{i,j}}{\text{d}t} & =f({𝐗}_{i,j}(t),{𝐙}_{j}(t),t;\theta_{i,j}),\\ \frac{\text{d}{𝐙}_{j}}{\text{d}t} & =h({𝒳}_{j}(t),{𝐙}_{j}(t),t;\Theta_{N_{j},j}), \end{array}$$ (6)

for $i=1,2,\ldots ,N_{j}$, and j=1,2,…,M. Here, ${𝐗}_{i,j}(t)$ is the state of the *i*th cell in the *j*th replicate having parameters $\theta_{i,j}$. For the *j*th replicate we have the subpopulation state, ${𝒳}_{j}(t)=[{𝐗}_{1,j}(t),{𝐗}_{2,j}(t),\ldots ,{𝐗}_{N_{j},j}(t)]$, the shared environment, ${𝐙}_{j}(t)$, and the subpopulation parameters $\Theta_{N_{j},j}=[\theta_{1,j},\theta_{2,j},\ldots ,\theta_{N_{j},j}]$.

Similarly, the observation process (Eq. (2)) becomes,


$$\begin{array}{c} {𝐘}_{i,j}(t)\sim g(\cdot |{𝐗}_{i,j}(t),\theta_{i,j},\eta ), \end{array}$$ (7)

where ${𝐘}_{i,j}(t)$ is the fluorescence of the *i*th cell in the *j*th replicate. The snapshot data for the *j*th replicate at time *t* > *t*_0_ is ${𝒴}_{j}(t)=[{𝐘}_{1,j}(t),{𝐘}_{2,j}(t),\ldots ,{𝐘}_{N_{j},j}(t)]$. We assume that *t*he groups of *M* replicates are observed a*t* the same *n* observations times $t_{1}<t_{2}<\cdots <t_{n}$, and denote $D_{j}=[{𝒴}_{j}(t_{1}),{𝒴}_{j}(t_{2}),\ldots ,{𝒴}_{j}(t_{n})]$ for the series of snapshots nominally labelled as the *j*th replicate, as in reality there are n×M replicates as the process of taking the snapshot typically destroys the sample. This leads to the complete flow cytometry dataset, including the set of snapshots in replicate subgroups, to be represented by $𝒟=[D_{1},D_{2},\ldots ,D_{M}]$.

Here, we assume that $\theta_{1,j},\theta_{2,j},\ldots ,\theta_{N_{j},j}$ are i.i.d. from a parametric distribution $p(\theta |\phi_{j})$ with subgroup hyper-parameter, $\phi_{j}$, representing the distribution parameters for the *j*th replicate, this subgroup hyper-parameter may also include the number of observed cells $N_{j}$ it this varies between replicates. Across all replicates, these hyper-parameters $\phi_{1},\phi_{2},\ldots ,\phi_{M}$, are distributed (i.i.d.) according to another parametric distribution, p(ϕ∣ψ) where ψ are the between group hyper-parameters (Fig 2).

#### Computational inference.

To quantify both inter-experimental and intra-experimental heterogeneity, characterised by p(·∣ψ) and $p(\cdot |\phi_{1}),p(\cdot |\phi_{2}),\ldots ,p(\cdot |\phi_{M})$, respectively, we aim to infer the hyper-parameters, $\psi ,\phi_{1},\phi_{2},\ldots \phi_{M}$. That is,


$$\begin{array}{c} p(\psi ,\phi_{1},\ldots \phi_{M}|𝒟)\propto p(𝒟|\psi ,\phi_{1},\ldots \phi_{M})p(\psi ,\phi_{1},\ldots \phi_{M}), \end{array}$$ (8)

where the joint prior that enforces the hierarchical structure of the model is given by


$$\begin{array}{c} p(\psi ,\phi_{1},\ldots \phi_{M})=p(\psi )\prod_{j=1}^{M}p(\phi_{j}|\psi ), \end{array}$$ (9)

and the likelihood is


$$\begin{array}{c} p(𝒟|\psi ,\phi_{1},\ldots \phi_{M})=\prod_{j=1}^{M}\left\{\int_{\Theta^{N}}p(D_{j}|\Theta_{N_{j},j})p_{j}(\Theta_{N_{j},j}|\phi_{j})\left[\prod_{i=1}^{N_{j}}\;\text{d}\theta_{i,j}\right]\right\}, \end{array}$$ (10)

with $p_{j}(\Theta_{N_{j},j}|\phi_{j})=\prod_{i=1}^{N_{j}}p(\theta_{i,j}|\phi_{j})$. In Eq. (10) we integrate out the individual cell parameters, $\theta_{i,j}$, within the likelihood function. This leads to a complex likelihood evaluation while reducing the dimension of the inference problem substantially to the space of hyper-parameters $\Phi^{M}\times \Psi$.

To maintain the lower dimensional inference problem from Eqs. (8)–(10) while avoiding the the direct likelihood calculation (10), we adopt an Approximate Bayesian computation (ABC) approach [47–49]. That is, we approximate Eq. (8) using the ABC posterior,


$$\begin{array}{c} p_{\text{ABC}}(\psi ,\phi_{1},\ldots ,\phi_{M}|𝒟)\propto \mathbb{P}(\rho (𝒟,{𝒟}_{s})\leq \epsilon |\phi_{1},\ldots ,\phi_{M})p(\phi_{1},\ldots ,\phi_{M}|\psi )p(\psi ). \end{array}$$ (11)

where ${𝒟}_{s}$ is simulated data and $\rho (𝒟,{𝒟}_{s})$ is a distribution matching discrepancy metric based on the Anderson-Darling distance,


$$\begin{array}{c} \rho (𝒟,{𝒟}_{s})=\frac{1}{M}\sum_{j=1}^{M}\sum_{k=1}^{n}A({𝒴}_{j}(t_{k}),{𝒴}_{j}^{s}(t_{k})), \end{array}$$ (12)

where ${𝒴}_{j}(t_{k})=[Y_{1,j}(t_{k}),Y_{2,j}(t_{k}),\ldots ,Y_{N,j}(t_{k})]$ is the real data snapshot for replicate *j* at time $t_{k}$, and ${𝒴}_{j}^{s}(t_{k})=[Y_{1,j}^{s}(t_{k}),Y_{2,j}^{s}(t_{k}),\ldots ,Y_{N,j}^{s}(t_{k})]$ is the simulated data snapshot for replicate *j* at time $t_{k}$. Here, the function A(𝒳,𝒴) is the Anderson-Darling distance,


$$\begin{array}{c} A(𝒳,𝒴)={\left[-N-\sum_{i=1}^{N}\frac{2i-1}{N}\log\left({\hat{F}}_{𝒳}\left(Y_{i}\right)\right)+\log\left(1-{\hat{F}}_{𝒳}\left(Y_{N+1-i,j}\right)\right)\right]}^{1/2}, \end{array}$$ (13)

where $𝒳=\{X_{1},X_{2},\ldots_{N}\}$ and $𝒴=\{Y_{1},Y_{2},\ldots ,Y_{N}\}$ are two sample sets and ${\hat{F}}_{𝒳}(x)$ is the empirical cumulative distribution for 𝒳. In this setting, we have that $p_{\text{ABC}}(\phi_{1},\phi_{2},\ldots ,\phi_{M},\psi |𝒟)\to p(\psi ,\phi_{1},\phi_{2},\ldots \phi_{M}|𝒟)$ as ϵ→0. We use an adaptive ABC scheme based on sequential Monte Carlo methods of Drovandi and Pettitt [50] that refines ϵ through sequential importance resampling (S2 Appendix).

## Results

To demonstrate the advantages of our approach, we perform simulations that represent *in silico* repeats of the experiments described in the Flow cytometry data Section based on published data [29,39,40]. The simulation setting enables experimental heterogeneity to be directly controlled and results to be compared to a known ground truth.

### Nano-engineered particle-cell interaction experiments

Murphy et al., [40] consider an ODE model of particle-cell interactions (See Example 1: Particle-cell interaction model) with each cell, i=1,2,…,N, having its own particle association rate, $r_{i}$,and carrying capacity, $K_{i}$. In the pooled sample setting, they consider these parameters $\theta_{i}=(r_{i},K_{i})$ to be log-Normally distributed,


$$\begin{array}{c} r_{i}\sim LogNormal(\mu_{r},\sigma_{r}),\;\text{and}\;K_{i}\sim LogNormal(\mu_{K},\sigma_{K}). \end{array}$$ (14)

For ease of interpretation, Eq (14) is re-parameterised such that $𝔼\left[r_{i}\right]=\mu_{r}$, $\text{Var}\left[r_{i}\right]=\sigma_{r}^{2}$, $𝔼\left[K_{i}\right]=\mu_{K}$, and $\text{Var}\left[K_{i}\right]=\sigma_{K}^{2}$. For inference, this leads to a vector of hyper-parameters of interest $\phi =(\mu_{r},\sigma_{r},\mu_{K},\sigma_{K})$.

#### Synthetic data scenarios.

Using the particle-cell interation model (See Example 1: Particle-cell interaction model) and the parameter distributions (Eq. (14)) for $r_{i}$ and $K_{i}$, we generate *in silico* data based on the experimental data collected by Faria et al., [29]. We consider *M* = 3 experimental replicates each with *N* = 20,000 cells with simulated flow cytometry snapshots taken at *n* = 6 time points that are considered identically prepared technical replicates (*t*_1_ = 1 [hr], *t*_2_ = 2 [hr], *t*_3_ = 4 [hr], *t*_4_ = 8 [hr], *t*_5_ = 16 [hr], and *t*_6_ = 24 [hr]). Within this setting, we generate synthetic flow cytometry data for the following three scenarios:

- Scenario 1: All *M* experimental replicates are i.i.d. with hyper-parameters $\mu_{r}=3.86125\times 10^{-7}$, $\sigma_{r}=4.56962\times 10^{-7}$, $\mu_{K}=10$, and $\sigma_{K}=2$.
- Scenario 2: Cell parameters in experimental replicate 1 are generated with a larger mean association rate of $\mu_{r}=9.0\times 10^{-7}$. Experimental replicates 2 and 3 are identical to Scenario 1.
- Scenario 3: Cell parameters in experimental replicate 1 are generated with a larger mean association rate $\mu_{r}=9.0\times 10^{-7}$ and cell parameters in experimental replicate 2 are generated with a larger mean association rate of $\mu_{r}=9.0\times 10^{-7}$ and a larger mean carrying capacity $\mu_{K}=20$. Experimental replicate 3 is identical to Scenario 1.

In all three scenarios we use, *c* = 1, $s=5.31\times 10^{-5}$, $v=1.01\times 10^{-6}$, and $u_{0}=9.95\times 10^{7}$. The specific values for these parameters are based on prior work of Murphy et al., [40].

**Fig 3:**
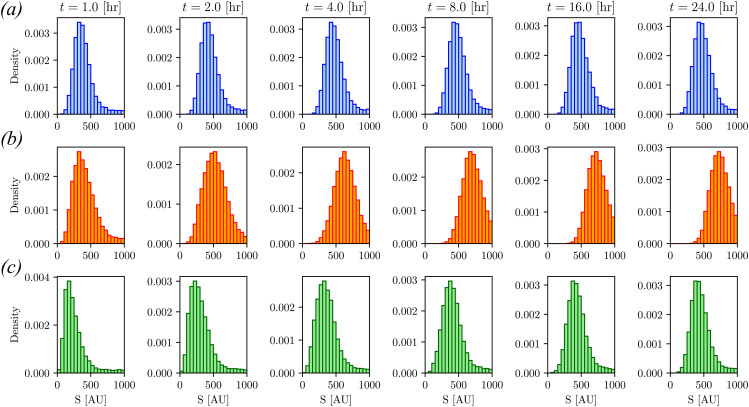
Synthetic flow cytometry population snapshot data example simulating nano-particle cell interaction data. Each histogram represents the simulated fluorescence *S* [AU] (that is Arbitrary Units) distribution for 20,000 observed cells interacting with nano-particles at one of six observation times (*t*_1_ = 1 [hr], *t*_2_ = 2 [hr], *t*_3_ = 4 [hr], *t*_4_ = 8 [hr], *t*_5_ = 16 [hr], and *t*_6_ = 24 [hr]). Replicate 1 (row **(a)**, blue) is generated with a mean particle association rate of $9.0\times 10^{-7}$ and a mean carrying capacity of $\mu_{K}=10$. Replicate 2 (row **(b)**, orange) is generated with a mean particle association rate of $3.86125\times 10^{-7}$ and a mean carrying capacity of $\mu_{K}=20$. Replicate 3 (row **(c)**, green) is generated with a mean particle association rate of $3.86125\times 10^{-7}$ and a mean carrying capacity of $\mu_{K}=10$. All replicates have the same standard deviation parameters for the association rate and carrying capacity of $\sigma_{r}=4.56962\times 10^{-7}$ and $\sigma_{K}=2$, respectively. Here, Replicate 2 (row (b), orange) represents an experimental outlier with higher carrying capacity, resulting in a distinctly larger fluorescence distribution mode at later times than Replicates 1 (row (a), blue) and 3 (row (c), green). Replicate 3 (row (c), green) represents an experimental outlier with a lower association rate, resulting in heavier skewness to the right than Replicates 1 (row (a), blue) and 2 (row (b), orange).

We can consider Scenario 1 as the ideal experimental conditions for pooled sample analysis, whereas Scenarios 2 and 3 represent different ways the pooled assumptions may not hold. We note that the variation we have introduced to these parameters is fairly modest. Fig 3 provides an example of the synthetic flow cytometry data generated for Scenario 3. The larger mean association rate of replicate 1 (Fig 3(a)) leads to a subtle increase in the location of the peak fluorescence at early times, t≤4[hr], compared with replicate 3 (Fig 3(c), the basis of Scenario 1), though at later times, *t* > 8 [hr], replicates 1 and 3 come into alignment. Replica*t*e 2 generally is more diffuse than replicates 1 and 3 for all times, with the peak fluorescence approaching a higher steady state due to the higher mean carrying capacity (Fig 3(b)).

#### The effect of pooling on quantification of heterogeneity.

**Fig 4:**
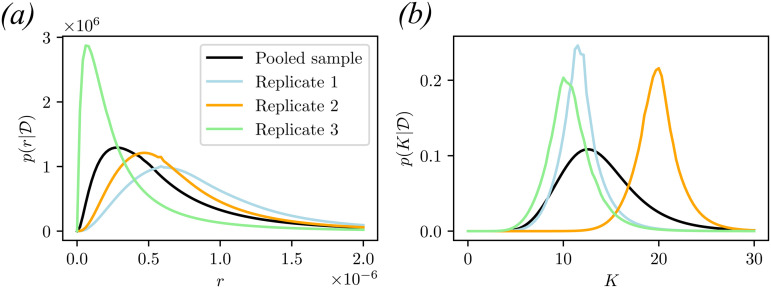
Heterogeneity in posterior cell parameter distributions resulting from pooled and hierarchical analysis of the particle-cell interaction model. Marginal distributions are shown for: **(a)** the particle association rate, *r*, and **(b)** the carrying capacity, *K*. Data Scenario 3 is used for this analysis (See Synthetic data scenarios and Fig 3). Here, Replicate 2 (orange) represents an experimental outlier with higher mean carrying capacity and Replicate 3 (green) represents an experimental outlier with a lower mean association rate. In this setting the pooled sample (black) does not adequately characterise the combined biological heterogeneity across experimental replicates.

Before presenting details of the pooled and hierarchical analysis, we focus on estimated distributions of the individual cell parameters resulting from this analysis. In particular, we consider the heterogeneity in the particle association rate (Fig 4(a)) and carrying capacity (Fig 4(b)) that results from the analysis of synthetic data Scenario 3 (See Synthetic data scenarios and Fig 3). This scenario captures a reasonable experimental setting for which the pooled i.i.d. assumptions are invalid due to experimental heterogeneity that is a confounding factor for the biological heterogeneity.

The pooled analysis adequately captures the variability in the cell parameter distributions with support that covers the bulk of each individual replicate distribution (Fig 4). However, the hierarchical analysis highlights that the actual biological heterogeneity is more complex with replicate 3 having a lower particle association rate (Fig 4(a)) and replicate 2 having larger carrying capacity (Fig 4(b)). These replicate specific distributions are not estimated independently, but rather, the hierarchical modelling structure allows for information sharing between replicates to provide quantification of experimental heterogeneity.

#### Pooled sample analysis.

We investigate the sensitivity of a pooled sample analysis to the i.i.d. assumptions. Following the pooled sample analysis protocol of Murphy et al., [40], we estimate the hyper-parameters, $\phi =(\mu_{r},\sigma_{r},\mu_{K},\sigma_{K})$, for each of the three synthetic data scenarios. These hyper-parameters characterise the heterogeneity (Eq. (14)) in particle association rates and carrying capacities throughout the cell population, given the cell population dynamics under the model of Murphy et al., [40] (See Example 1: Particle-cell interaction model). The Bayesian analysis follows the approach described in the Statistical framework Section, using independent uniform priors, $\mu_{r}\sim 𝒰(0,10^{-6})$, $\mu_{K}\sim 𝒰(0,50)$, $\sigma_{r}\sim 𝒰(0,2\times 10^{-6})$, and $\sigma_{K}\sim 𝒰(0,10)$. These priors are consistent with the analysis of Murphy et al., [40], and cover a range of physically viable values.

**Fig 5:**
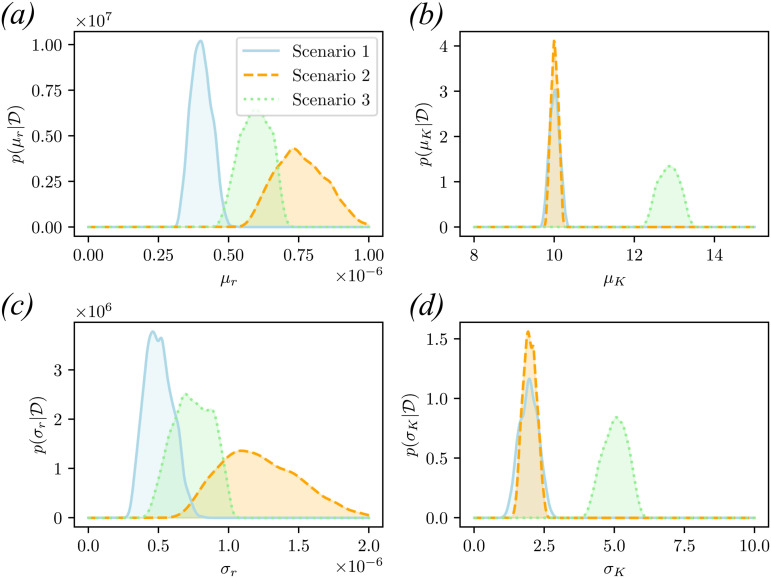
Pooled sample analysis of the nano-engineered particle-cell interaction experiment for the three synthetic data scenarios. As described in Synthetic data scenarios, Scenario 1 (blue) is the i.i.d. case with no outliers, Scenario 2 (orange) has a single outlier replicate with a larger mean association rate, and Scenario 3 (green) has an additional outlier with a larger mean carrying capacity. Marginal posterior distributions for the hyper-parameters: **(a)** mean association rate, $\mu_{r}$; **(b)** mean carrying capacity, $\mu_{K}$; **(c)** association rate standard deviation, $\sigma_{r}$; and **(d)** carrying capacity standard deviation, $\sigma_{K}$.

The results, as shown in Fig 5, indicate that the estimated hyper-parameters are sensitive to the synthetic data scenario. Firstly, the analysis of Scenario 1 results in posterior distributions that accurately recover the true hyper-parameters of $\mu_{r}=3.86125\times 10^{-7}$ (Fig 5(a)), $\mu_{K}=10$ (Fig 5(b)), $\sigma_{r}=4.56962\times 10^{-7}$ (Fig 5(c)), and $\sigma_{K}=2$ (Fig 5(d)). This is expected, since Scenario 1 represents our well-specified setting in which each replicate contains identical populations of cells. While this simulation validates the use of the pooled approach when when experimental replicates are genuinely the same [39,40], the results for Scenarios 2 and 3 demonstrate substantial sensitivity of the posteriors given deviations in one of the replicates.

In Scenario 2, only one replicate was modified to have a mean association rate of $\mu_{r}=9.0\times 10^{-7}$ resulting in potential outlier in one of the replicates (Fig 3(a)) compared with the unmodified replicates (Fig 3(c)). This outlier replicate strongly affects the estimates for both the pooled associate rate distributions with inflated means $\mu_{r}$ (Fig 5(a)) and standard deviations $\sigma_{r}$ (Fig 5(c)). While the results represent the best pooled sample inference given the outlier replicate, the results may not be meaningful for prediction in biological applications since the inferred heterogeneity does not represent any of the replicates accurately. The results are even more concerning for Scenario 3, where an addition replicate is modified to be an outlier association rate, $\mu_{r}=9.0\times 10^{-7}$, and in carrying capacity $\mu_{K}=20$, since a shift in all four hyper-parameters occurs (Fig 5). This means that the heterogeneity estimates for the pooled population are not representative of any of the three replicate populations. These results motivate the adoption of our extended hierarchical model in experimental settings for which variation is expected between replicates.

#### Hierarchical analysis.

We now demonstrate the advantages of our hierarchical framework (See Statistical framework) as a method for capturing heterogeneity in flow cytometry data when variation between replicates is present. We consider the same synthetic data scenarios (See Synthetic data scenarios) and apply our hierarchical framework to the particle-cell interaction model (See Example 1: Particle-cell interaction model) of Murphy [40]. Specifically, we consider the setting where the *i*th cell in the *j*th replicate population has its own associated rate, $r_{i,j}$, and carrying capacity, $K_{i,j}$. Following the pooled sample setting, we assume these parameters $\theta_{i,j}=(r_{i,j},K_{i,j})$ are log-Normally distributed, however, we deviate from the pooled assumption by allowing each replicate population j=1,2,…,M to have its own mean association rate, $\mu_{r,j}$, and mean carrying capacity, $\mu_{K,j}$,


$$\begin{array}{c} r_{i,j}\sim LogNormal(\mu_{r,j},\sigma_{r}),\;\text{and}\;K_{i,j}\sim LogNormal(\mu_{K,j},\sigma_{K}). \end{array}$$

Note that the pooled sample model is recovered for the special case where $\mu_{r,1}=\mu_{r,2}=\cdots =\mu_{r,M}$ and $\mu_{K,1}=\mu_{K,2}=\cdots =\mu_{K,M}$. We further assign a Normal distribution to the replicate population means,


$$\begin{array}{c} \mu_{r,j}\sim 𝒩(m_{r},s_{r}),\;\text{and}\;\mu_{K,j}\sim 𝒩(m_{K},s_{K}), \end{array}$$

where $m_{r}$ (resp. $m_{K}$) and $s_{r}$ (resp. $s_{K}$) are the mean and standard deviation of the replicate population association rate (resp. carrying capacity) means. Thus we infer intra-experimental heterogeneity for replicate population *j* through inference of the hyper-parameters $\phi_{j}=(\mu_{r,j},\mu_{K,j},\sigma_{r},\sigma_{K})$ and inter-experimental heterogeneity though the population level hyper-parameters, $\psi =(m_{r},m_{K},s_{r},s_{K})$.

We perform Bayesian inference for the full joint posterior for the two levels of hyper-parameters (Eqs. (8) and (11)). For the population level parameters, we apply independent uniform priors for the means $m_{r}\sim 𝒰(0,10^{-6})$, $m_{K}\sim 𝒰(0,50)$, and weakly informative half Cauchy priors for the standard deviations $\sigma_{r}\sim \text{Half-Cauchy}(3\times 10^{-7})$ and $\sigma_{k}\sim \text{Half-Cauchy}(0.5)$. Note the shape parameters for the half Cauchy priors are determined based on the guidelines of Gelman et al., [51] with regard to selection of weakly informative priors for standard deviation terms in Bayesian hierarchical models with small numbers of groups.

**Fig 6:**
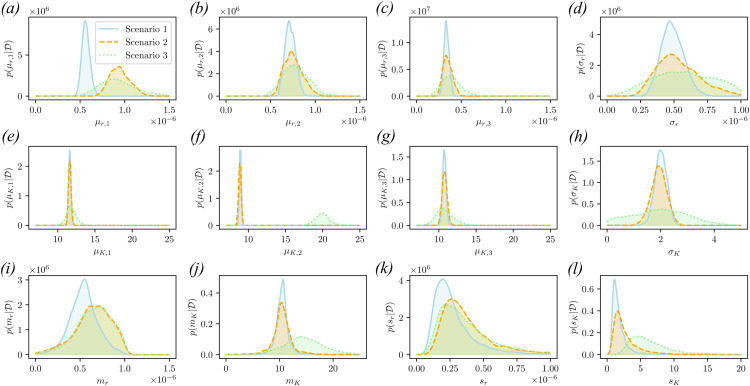
Hierarchical analysis of the nano-engineered particle-cell interaction experiment for the three synthetic data scenarios. As described in Synthetic data scenarios, Scenario 1 (blue) is the i.i.d. case with no outliers, Scenario 2 (orange) has a single outlier replicate with a larger mean association rate, and Scenario 3 (green) has an additional outlier with a larger mean carrying capacity. Marginal posterior distributions are shown for the replicate hyper-parameters: **(a)**–**(c)** mean association rates, $\mu_{r,j}$ for replicates *j* = 1,2,3; **(d)** association rate standard deviation, $\sigma_{r}$; **(e)**–**(g)** mean carrying capacity, $\mu_{K,j}$ for replicates *j* = 1,2,3; and **(h)** carrying capacity standard deviation, $\sigma_{K}$. Marginal posterior distribution are shown for the population level hyper-parameters: **(i)** mean of association rate means, $m_{r}$; **(j)** mean of carrying capacity means, $m_{K}$; **(k)** standard deviation of association rate means, $s_{r}$; and **(l)** standard deviation of carrying capacity means, $s_{K}$. Outlier mean association rates ((a), orange and green) and mean carrying capacity ((f), green) are identified, whereas information sharing lead to robust population level heterogeneity estimates (i)–(l).

The results, shown in Fig 6, highlight the value of our hierarchical approach. In Scenario 1 we accurately identify replicates are i.i.d. with $p(\mu_{r,1}|𝒟)\approx p(\mu_{r,2}|𝒟)\approx p(\mu_{r,3}|𝒟)$ (Fig 6(a)–6(c)), and $p(\mu_{K,1}|𝒟)\approx p(\mu_{K,2}|𝒟)\approx p(\mu_{K,3}|𝒟)$ (Fig 6(e)–6(g)). Furthermore the outlier replicates in Scenario 2 (replicate 1 with larger $\mu_{r,1}$; Fig 6(a)) and Scenario 3 (replicate 1 with larger $\mu_{r,1}$, and replicate 2 with larger $\mu_{K,2}$; Fig 6(a) and 6(f)), are directly captured with only minor inflation in the variance of the posterior distributions for the remaining replicate populations (Fig 6(b) and 6(c) for Scenario 2, and Fig 6(b), 6(c), 6(e) and 6(g) for Scenario 3).

In addition to reliable characterisation of heterogeneity for each replicate population, we also obtain estimates of the inter-experimental heterogeneity that are insensitive to variation in a single replicate. This is evidenced by substantial overlap in the posterior probability densities around the true population level means (Fig 6(i) and 6(j)) and standard deviations (Fig 6(k) and 6(l)). In almost all cases, the true population level hyper-parameter is within the bulk density region of the posterior distribution. The exception, is the population standard deviation parameter for the carrying capacity mean $s_{K}$ in Scenario 3 (Fig 6(l)), however, the deviation in the posterior distributions for $s_{K}$ across scenarios is substantially less than that of the equivalent pooled sample analysis (Fig 5(d)). This robustness in population level inferences arises from the between-replicate information sharing inherent to the hierarchical model.

### Dual-labelled probe internalisation experiments

To demonstrate the generality of or framework, we perform a similar simulation study based on the study of heterogeneity in cell internalisation processes by Browning et al., [39]. The internalisation process of a cell is modelled according an ODE system (See Example 2: Internalisation model) with each cell having its own internalisation rate, $\lambda_{i}$, recycling rate, $\beta_{i}$, and initial receptor density $R_{i}$. The heterogeneity in these parameters is modelled, in the pooled sample setting, according to


$$\begin{array}{cc} \lambda_{i} & \sim \text{ShiftedGamma}(\mu_{\lambda },\sigma_{\lambda },\omega_{\lambda }),\\ \beta_{i} & \sim \text{ShiftedGamma}(\mu_{\beta },\sigma_{\beta },\omega_{\beta }),\\ R_{0,i} & \sim \text{ShiftedLogNormal}(\mu_{R_{0}},\sigma_{R_{0}}), \end{array}$$

where $\mu_{\lambda }$, $\sigma_{\lambda }$, and $\omega_{\lambda }$ (resp. $\mu_{\beta }$, $\sigma_{\beta }$, and $\omega_{\beta }$) are the mean, standard deviation, and skewness of the internalisation rate (resp. recycling rate). Here, $\mu_{R_{0}}$, $\sigma_{R_{0}}$ are the standard log-Normal parameters and the distribution of *R*_0,*i*_ is shifted such that $𝔼\left[R_{0,i}\right]=1$ (See Browning et al., [39] for further details). For inference, these distribution hyper-parameters are of interest along with the disassociation rate *p*, that is, $\phi =(\mu_{\lambda },\sigma_{\lambda },\omega_{\lambda },\mu_{\beta },\sigma_{\beta },\omega_{\beta },\mu_{R_{0}},\sigma_{R_{0}},p)$. As per Eq. (5), parameters for to the observation process, $\eta =(\alpha_{1},\alpha_{2},\sigma_{1},\sigma_{2})$, are also inferred, however, we do not focus on these here.

#### Synthetic data scenarios.

For the internalisation model (See Example 2: Internalisation model) we generate synthetic data inspired by the data collected by Browning et al., [39] (See Dual-labelled probe internalisation data). Here, we have *M* = 3 replicates, each consisting of quenched/unquenched sample pairs of *N* = 1,000 cells each. Simulated flow cytometry snapshots are taken at *n* = 7 observation times, *t*_1_ = 5 [min], *t*_2_ = 10 [min], *t*_3_ = 20 [min], *t*_4_ = 30 [min], *t*_5_ = 40 [min], *t*_6_ = 120 [min], and *t*_7_ = 180 [min]. As before (See Synthetic data scenarios), we treat the different snapshots as being generated by identically prepared technical replicates, and thus having the same intra-experimental heterogeneity. We consider two scenarios based:

- Scenario 1: All *M* replicates are i.i.d., with hyper-parameters $\mu_{\lambda }=0.08$, $\sigma_{\lambda }=0.01$, $\omega_{\lambda }=0.9$, $\mu_{\beta }=0.05$, $\sigma_{\beta }=0.005$, $\omega_{\beta }=-1.0$ $\mu_{R}=0.05$, and $\sigma_{R}=0.01$.
- Scenario 2: Cell parameters in replicate 1 are generated with a smaller mean internalisation rate $\mu_{\lambda }=0.02$. Replicates 2 and 3 are identical to Scenario 1.

**Fig 7:**
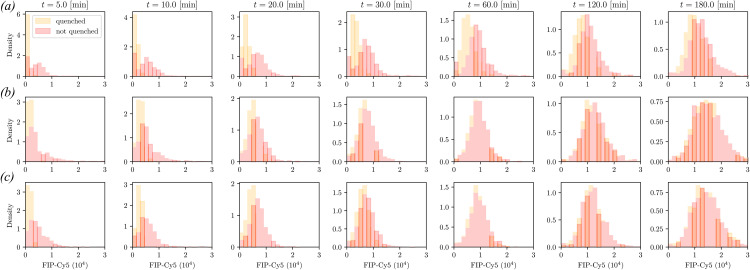
Synthetic flow cytometry population snapshot data example simulating the dual-labelled probe internalisation experiments. Each histogram represent the simulated fluorescence distribution of the quenchable probe (FIP-Cy5) for 1,000 observed cells cells in the quenched sample (orange) and 1,000 observed cells in the unquenched sample (red) at one of seven observation times (*t*_1_ = 5 [min], *t*_2_ = 10 [min], *t*_3_ = 20 [min], *t*_4_ = 30 [min], *t*_5_ = 60 [min], *t*_6_ = 120 [min], and *t*_7_ = 180 [min]). Replicate 1 (row **(a)**) is generated with a mean internalisation rate of $\mu_{\lambda }=0.02$, a mean recycling rate of $\mu_{\beta }=0.05$ and a log-normal mean initial receptor concentration of $\mu_{R}=0.05$. Replicates 2 and 3 (rows **(b)** and **(c)**) are generated with a mean internalisation rate of $\mu_{\lambda }=0.08$, a mean recycling rate of $\mu_{\beta }=0.05$ and a log-normal mean initial receptor concentration of $\mu_{R}=0.05$. All replicates have the same standard deviation parameters, $\sigma_{\lambda }=0.01$, $\sigma_{\beta }=0.01$, and $\sigma_{R}=0.01$. Here Replicate 1 (row (a)) represents an experimental outlier with a lower mean internatisation rate, resulting in a clear differences between quenched (orange) and unquenched (red) fluorescent distributions even at late time. In contrast, both Replicates 2 and 3 (rows (b) and (c)), obtain near identical quenched (orange) and unquenched (red) fluorescent distributions after *t* = 60 [min].

In both scenarios, *p* = 0.075, $\alpha_{1}=8500$, $\alpha_{2}=38$, $\sigma_{1}=5$, and $\sigma_{2}=2$. Parameter values are obtained based on the analysis of Browning et al., [39]. Fig 7 provides an example of the synthetic flow cytometry data generated for Scenario 2.

As with the nano-particle cell interaction example (See Synthetic data scenarios), Scenario 1 represents the setting in which the pooled analysis is expected to be accurate, whereas Scenario 2 includes an outlier replicate (Fig 7(a)) with a smaller internalisation rate. This results in the quenched and unquenched samples being distinguishable throughout all snapshots. By comparison, replicates 2 and 3 lead to almost identical fluorescence distributions for quenched and unquenched at *t* = 30 [min].

#### The effect of pooling on quantification of heterogeneity.

**Fig 8:**
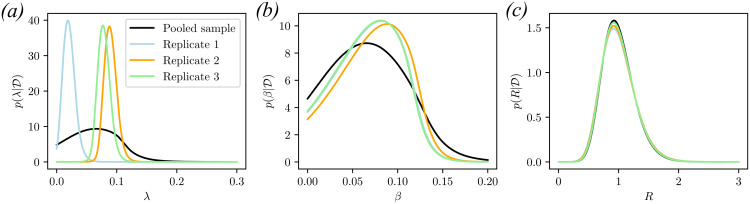
Heterogeneity in posterior cell parameter distributions resulting from pooled and hierarchical analysis of the internalisation model. Marginal distributions are shown for: **(a)** the internalisation rate, λ, **(b)** the recycling rate, β, and **(c)** the initial receptor density, *R*. Data Scenario 2 is used (See Synthetic data scenarios and Fig 7) which exhibits variation across replicates in the mean internalisation rate. Here, Replicate 1 (blue) represents an experimental outlier with a lower mean internalisation rate (a). In this setting, the pooled sample (black) is not able to capture the biological heterogeneity in interalisation rates (a) or recycling rates (b). The pooled sample is able to quantify biological heterogeneity in the initial receptor density (c) as this parameter is insensitive to the rate parameters.

In the pooled analysis, we note that the estimated variance of the internalisation rate is inflated to account for the variation across experimental replicates (Fig 8(a)). While the hierarchical approach provides more details with replicate specific parameter distributions, the inflated variance in the pooled approach seems an appropriate global approximation. However, the pooled analysis also inflates the variance of the recycling rate (Fig 8(b)) for which there is no substantial experimental heterogeneity. Conversely the pooled and hierarchical results for the initial receptor density are practically identical (Fig 8(c)). A reasonable explanation for the incorrect inflation of the β distribution is the relationship between λ and β in the model Eq. (4) as receptor recycling is effectively a reversal of internalisation.

#### Pooled sample analysis.

Here we investigate how deviations in the i.i.d. assumption affect quantification of biological heterogeneity, and the impact this has on estimates of global parameters, such as the disassociation probability *p*. For the pooled sample analysis we follow Browning et al., [39] to estimate the hyper-parameters, $\phi =(\mu_{\lambda },\sigma_{\lambda },\omega_{\lambda },\mu_{\beta },\sigma_{\beta },\omega_{\beta },,\mu_{R_{0}},\sigma_{R_{0}},p)$ that relate to heterogeneity in internalisation rates, recycling rates, and the receptor concentrations. In addition, the purely chemical disassociation probability constant *p* is also.

**Fig 9:**
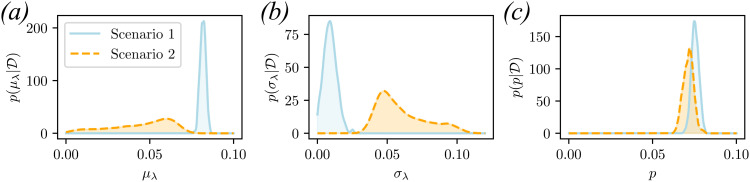
Pooled sample analysis of the dual-label probe internalisation experiment for the two synthetic data scenarios. As described in Synthetic data scenarios, Scenario 1 (blue) is the i.i.d. case with no outliers, and Scenario 2 (orange) has a single outlier replicate with a lower mean internalisation rate. Marginal posterior distributions for the hyper-parameters: **(a)** mean internalisation rate, $\mu_{\lambda }$; **(b)** internalisation rate standard deviation, $\sigma_{\lambda }$; and **(d)** the disassociation rate, *p*.

The experimental heterogeneity incorporated into data Scenario 2 results in substantial estimated bias and variance inflation for both the mean (Fig 9(a)) and standard deviation (Fig 9(b)) parameters for the internalisation rate. Just as with the particle-cell interaction model (See Nano-engineered particle-cell interaction experiments), the pooled sample analysis results are extremely sensitive to experimental heterogeneity. In addition, inferences of parameters with no biological or experimental heterogeneity, such as the disassociation rate *p* (Fig 9(c)), are impacted by experimental heterogeneity in the internalisation rate. Other parameter inferences are also inaccurate for Scenario 2, however, we focus here on the internalisation rate and the disassociation rate.

#### Hierarchical analysis.

Expanding the analysis of Browning et al., [39] according to our framework (See Statistical framework) leads to the *i*th cell in the *j*th replicate population having its own internalisation rate, $\lambda_{i,j}$, recycling rate, $\beta_{i,j}$, and initial receptor density, $R_{i,j}$. Further we allow the each of the replicate populations to have its own mean internalisation rate, $\mu_{\lambda ,j}$, mean recycling rate, $\mu_{\beta ,j}$, and mean initial receptor density, $\mu_{R,j}$,


$$\begin{array}{cc} \lambda_{i,j} & \sim \text{ShiftedGamma}(\mu_{\lambda ,j},\sigma_{\lambda },\omega_{\lambda }),\\ \beta_{i,j} & \sim \text{ShiftedGamma}(\mu_{\beta ,j},\sigma_{\beta },\omega_{\beta }),\\ R_{i,j} & \sim \text{ShiftedLogNormal}(\mu_{R,j},\sigma_{R}), \end{array}$$

and treat the replicate population means as normally distributed,


$$\begin{array}{c} \mu_{\lambda ,j}\sim 𝒩(m_{\lambda },s_{\lambda }),\;\mu_{\beta ,j}\sim 𝒩(m_{\beta },s_{\beta }),\;\text{and}\;\mu_{R_{0},j}\sim 𝒩(m_{R_{0}},s_{R_{0}}). \end{array}$$

**Fig 10:**
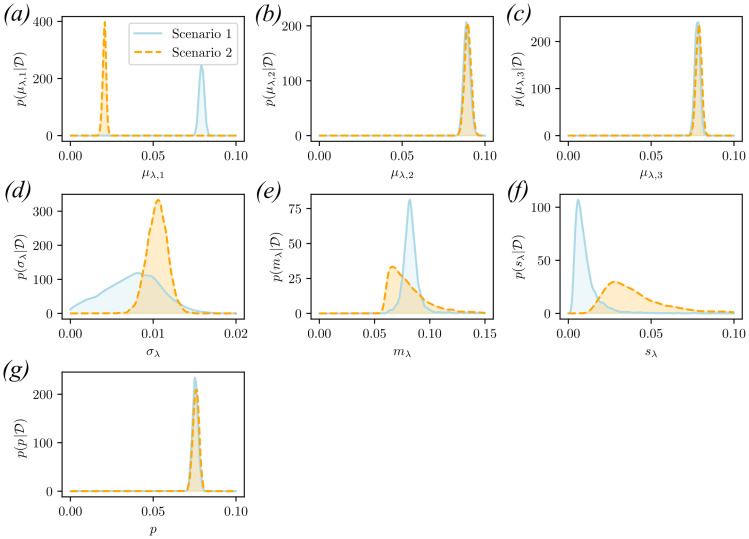
Hierarchical analysis of the dual-labelled probe internalisation experiment for the two synthetic data scenarios. As described in Synthetic data scenarios, Scenario 1 (blue) isn the i.i.d. cae with no outliers, and Scenario 2 (orange) has a singe outlier replicate with a lower mean internalisation rate. Marginal posterior distributions are shown for the replicate hyper-parameters: **(a)**–**(c)** mean internalisation rates, $\mu_{\lambda ,j}$ for replicates *j* = 1,2,3; **(d)** internalisation rate standard deviation, $\sigma_{\lambda }$. Marginal posterior distributions are shown for the population level hyper-parameters: **(e)** mean of internalisation rate means, $m_{\lambda }$; **(f)** standard deviation of internalisation rate means, $s_{\lambda }$; and **(g)** disassociation rate *p*. The outlier mean internalisation rate in Scenario 2, Replicate 1 ((a), orange) is identified, while population estimates ((e)–(f)) and homogeneous disassociation rate estimates (g) are robust to this outlier.

We observe improved results that accurately estimate the mean internalisation rate for each replicate population in both scenarios (Fig 10(a)–10(c)) while quantifying the experimental heterogeneity across replicates (Fig 10(e)–10(f)). Furthermore the disassociation rate is precisely the same in both scenarios ((Fig 10(g)). These results highlight that pooled analysis should only be used when there is high certainty in the i.i.d. assumption across replicates.

## Discussion

The reliable quantification of heterogeneity in cell populations is crucial to ensure accurate assessment and prediction of treatment efficacy [12–14], especially in the setting of targeted therapeutics [9–11]. Various approaches to modelling and analysis have been developed in the literature to quantify this heterogeneity using cell population snapshots obtained through flow cytometry. However, variability between experimental replicates is rarely considered. In this work, we present a Bayesian hierarchical framework to quantify biological and experimental heterogeneity simultaneously when using mechanistic models of cell dynamics. This is an important development, since previous approaches for quantification of heterogeneity using mechanistic models have only considered pooled samples and neglected experimental heterogeneity [29,30,39,40].

We demonstrated our framework using a two-level hierarchical approach that modelled heterogeneity within experimental replicates and heterogeneity between each replicate. This could naturally be extended to include additional levels that might describe heterogeneity across different labs, instrument configurations, or cell lines. Alternatively, in other experimental settings that support tracking of individual cells [32,52], then the individual cell parameters could also be estimated. This would naturally come with additional computational challenges due to the larger dimensional parameter space, but such challenges are not insurmountable (S1 Appendix).

Our simulation examples assume the scenario in which experimental replicates are repeats of the same experimental conditions. That is, the inter-experimental heterogeneity would be largely to do with variations in the instrumental configuration, operator and variations in uncontrolled aspects of the environment. However, our tools are also entirely applicable to setting in which experimental conditions are also varied, such as different treatments, dosage levels, or cell perturbations [32,52]. As noted in S1 Appendix, such an extension could be introduced with a three-level hierarchical model. As a result, data integration over multiple experimental conditions could be exploited to reduce certain parameter identifiability problems that are common in single-cell experiments [32,53].

Here, we only consider inter-experimental heterogeneity in the parameter means across replicate populations. Experimental heterogeneity in the parameter variances are mathematically straightforward to introduce. However, some care should be taken to avoid non-identifiablity issues arising, especially for small numbers of replicates *M* [54]. Weakly informative priors, such as the Half-Cauchy prior, are necessary for the variance hyper-parameter of the sub-population parameter means in the low replicate number setting *M* = 3 [51], and this would necessarily be more complex with experimental heterogeneity in variances [55]. As a result, we recommend that experimental heterogeneity in the replicate population parameter variances only be considered when the number of replicates, *M*, is large.

As our method was developed and presented as an extension to the pooled sample approaches [39,40], we utilised similar modelling and analysis decisions. These could be adjusted as appropriate for other applications. For example, we assumed that heterogeneity is driven by variation between individual cells and that stochastic effects are negligible. In rare circumstances analytical solutions are available to account for non-negligible intrinsic noise [56], however, it is often necessary to use stochastic model [49,57]. Since we rely on ABC for parameter inference, our framework is completely applicable to stochastic modelling approaches, including agent-based models or stochastic differential equations (SDEs).

In other settings where intrinsic stochasticity is negligible and the population size is very large, continuum models (in the sense of the observed cell count $N_{j}\to \infty$ for all *j*) could be used [58]. For example population balance models or phenotype-structured partial differential equation models could be plausible in this setting may be appropriate and would enable more complex dynamics to be included, such as cell division and cell death [58–61]. To capture intra-experimental heterogeneity in this case, care must be taken as individual cells are no longer explicitly modelled [62], but rather a continuous cell population density. This renders biologically heterogeneous parameters to be functions of cell density [63]. In effect this renders biological heterogeneity as a random function, rather than $N_{j}$ i.i.d. samples $\theta_{1,j},\ldots ,\theta_{N_{j},j}\sim p(\cdot |\psi_{j})$. To characterise biological heterogeneity in this setting, distributions on functions or random measures [64–67] may be needed. This would be an interesting line of future research.

The use of traditional simulation-based inference methods, such as ABC, could be considered a limitation due to the necessity of a tolerance threshold, ϵ>0, that affects the accuracy of the posterior approximation [68–70]. In addition, targeting a very small ϵ is generally computationally burdensome and modern machine learning approaches are generally considered more efficient in this respect [70–73]. In the non-linear mixed-effects modelling setting, these methods have been extended to achieve more computationally efficient inference through the use of the amortization property to rapidly estimate individual scale likelihoods that can be adapted to obtain population level posteriors [35,36]. However, one desirable property of ABC, particularly in the context of biological modelling, is its robustness to model misspecification or model uncertainty [74–77]. While robust versions of modern machine learning methods continue to be developed [78–84], there is a serious lack of theoretical guarantees on how these methods behave in the misspecified setting. As a result, we still recommend ABC approaches when model misspecification is likely. In situations where ABC is completely computationally infeasible, then the methods of Arruda et al. [35], and Häggström [36] could potentially be extended to our modelling framework. Some care would be needed in dealing with our shared environment component (Eq. (6)), but this would be a promising step forward, especially if ideas from robust simulation-based inference could be incorporated [78–84].

While our approach is developed with flow cytometry experiments in mind [39,40], the method itself is general to any setting with population snapshot data taken from interacting individuals. For example, in the study of marine ecology, hierarchical Bayesian models are frequently used to describe coral reefs that vary across spatial regions [85,86], however, mechanistic descriptions are typically only done on the level of individual reefs [87,88]. Our approach would be well suited to account for spatial heterogeneity in these mechanistic models for more realistic large scale reef forecasts.

## Conclusion

We have highlighted that caution must be taken when using pooled sample approaches to avoid potential bias or inaccuracies in quantification of biological heterogeneity. Our approach provides a means of reliable inference when experimental heterogeneity is present. Furthermore, we can quantify the heterogeneity across experiential replicates. This, in turn, could be used to identify potentially problematic replicates and improve experimental protocols. Due to these properties, our method has the potential to greatly enhance the biological insights that can be obtained through flow cytometry and advance developments in quantitative biology. Finally, our approach is generally applicable to other experimental techniques, such as microarrays that can result in drift and change between experimental replicates [89], and to other analysis settings, such as the integration of multiple experimental conditions [32,52]. We provide open-source implementations of our methods on GitHub.

## Supporting information

S1 Appendix Three-level hierarchical extensions. (PDF)

S2 Appendix Computational inference. (PDF)
