# Supplementary material for: A multilevel hierarchical framework for quantification of experimental heterogeneity in population snapshot data
Source: PLoS Comput Biol. 2026 Jun 15;22(6):e1014379. doi: 10.1371/journal.pcbi.1014379 (PMC13286279; doi:10.1371/journal.pcbi.1014379)
Supplement: S1 Appendix — (PDF) [file pcbi.1014379.s001.pdf]

Supporting information for "A multilevel hierarchical framework for quantification of experimental heterogeneity"

David J. Warne[1*], Xiangrun Zhu[1], Thomas P. Steele[1], Stuart T. Johnston[2], Scott A. Sisson[3], Matthew Faria[4], Ryan J. Murphy[5], Alexander P. Browning[2,6]

1 School of Mathematical Sciences, Queensland University of Technology, Brisbane, Australia
2 School of Mathematics and Statistics, The University of Melbourne, Melbourne, Australia
3 School of Mathematics and Statistics, University of New South Wales, Sydney, Australia
4 Department of Biomedical Engineering, The University of Melbourne, Melbourne, Australia
5 School of Mathematical Sciences, Adelaide University, Adelaide, Australia
6 Mathematical Institute, University of Oxford, Oxford, United Kingdom

* david.warne@qut.edu.au

S1 Appendix. Extension to biological, technical, and experimental replicates

In the main manuscript, we consider a two layers of heterogeneity with details presented in the main manuscript. Here we will extend the approach to three levels heterogeneity that we will refer to as heterogeneity between *biological replicates*, *technical replicates*, and *experimental replicates*. Noting that terminology varies in the experimental biology literature, we will adopt the following terminology: each cell from a given cell line within a cell culture is a biological replicate; each cell culture population from a collection of identically prepared cell cultures is a technical replicate; and the reproduction of an entire experiment is an experimental replicate. Note that there is no requirement for experiments to be identically prepared, for example, each experiment could have a different treatment, cell-line, or genetic perturbation.

Suppose we adopt the random ODE approach from the main manuscript with L experiments with the kth experiment having M_k replicates cell populations. In the jth replicate, $N_{j,k}$ cells are analysed. Let $\mathbf{X}_{i,j,k}(t)$ and $\boldsymbol{\theta}_{i,j,k}$ be the state at time $t > t_0$ and parameters of the ith cell (biological replicate) within the jth cell culture (technical replicate) from the kth experiment (experimental replicate). We have the system evolution and observation process as,

$$\frac{\mathrm{d}\mathbf{X}_{i,j,k}}{\mathrm{d}t} = f(\mathcal{X}_{j,k}(t), t, ; \theta_{i,j,k})$$
$$\mathbf{Y}_{i,j,k}(t) \sim g(\cdot \mid \mathbf{X}_{i,j,k}(t), \theta_{i,j,k})$$

for $i = 1, 2, \ldots, N_{j,k}$, $j = 1, 2, \ldots, M_k$, and $k = 1, 2, \ldots, L$. For ease of notation, we exclude the shared environment and observation process parameters described in the main manuscript. The state of the jth sub-population from the kth experiment is $\mathcal{X}_{j,k}(t) = [\mathbf{X}_{1,j,k}(t), \mathbf{X}_{2,j,k}(t), \ldots, \mathbf{X}_{N_{j,k},j,k}(t)]$. The snapshot data for the jth technical replicate from the kth experiment at time $t > t_0$ is

$\mathcal{Y}_{j,k}(t) = [\mathbf{Y}_{1,j,k}(t), \mathbf{Y}_{2,j,k}(t), \dots \mathbf{Y}_{N_{j,k},j,k}(t)]$. Then for n observation times, $t_1 < t_2 < \cdots < t_n$, the jth technical replicate from the k experiment is $D_{j,k} = [\mathcal{Y}_{j,k}(t_1), \mathcal{Y}_{j,k}(t_2), \mathcal{Y}_{j,k}(t_n)]$. Then the kth experiment set of snapshots is $\mathcal{D}_k = [D_{1,k}, D_{2,k}, \dots, D_{M_k,k}]$. Finally the complete dataset, containing all biological, technical and experimental replicates is, $\mathfrak{D} = [\mathcal{D}_1, \mathcal{D}_2, \dots, \mathcal{D}_L]$.

Biological heterogeneity is modelled by $\boldsymbol{\theta}_{1,j,k}, \boldsymbol{\theta}_{2,j,k}, \dots, \boldsymbol{\theta}_{N_{j,k},j,k} \overset{\text{i.i.d.}}{\sim} p(\cdot \mid \boldsymbol{\phi}_{j,k})$ with each sub-population having its own population hyper-parameters $\boldsymbol{\phi}_{j,k}$. The heterogeneity in these hyper-parameters, that is technical heterogeneity, is modelled according to $\boldsymbol{\phi}_{1,k}, \boldsymbol{\phi}_{2,k}, \dots, \boldsymbol{\phi}_{M_k,k} \overset{\text{i.i.d.}}{\sim} p(\cdot \mid \boldsymbol{\psi}_k)$ where $\boldsymbol{\psi}_k$ is the experimental parameters for the kth experiment. Finally we have experimental heterogeneity given by $\boldsymbol{\psi}_1, \boldsymbol{\psi}_2, \dots, \boldsymbol{\psi}_L \overset{\text{i.i.d.}}{\sim} p(\cdot \mid \xi)$ with $\xi \in \boldsymbol{\Xi}$ representing hyper-parameters charaterising the variation between experiments.

Just as is the main manuscript, estimating individual cell parameters $\boldsymbol{\theta}_{i,j,k}$ is considered meaningless, thus given data $\mathfrak{D}$ the aim is to infer the three levels of hyper parameters representing: experimental heterogeneity ξ; technical heterogeneity $\boldsymbol{\psi}_k$ for $k = 1, 2, , \dots, L$; and biological heterogeneity, $\boldsymbol{\phi}_{j,k}$ for $j = 1, 2, \dots, M_k$, and $k = 1, 2, \dots, L$. This leads to the inference problem,

$$p(\xi, \boldsymbol{\psi}_{1:L}, \boldsymbol{\phi}_{1:M_k,1:L} \mid \mathfrak{D}) \propto p(\mathfrak{D} \mid \xi, \boldsymbol{\psi}_{1:L}, \boldsymbol{\phi}_{1:M_k,1:L}) p(\xi, \boldsymbol{\psi}_{1:L}, \boldsymbol{\phi}_{1:M_k,1:L}), \tag{1}$$

where $\boldsymbol{\phi}_{1:M_k,1:L} = [\boldsymbol{\phi}_{1,1}, \boldsymbol{\phi}_{2,1}, \dots, \boldsymbol{\phi}_{M_k,1}, \boldsymbol{\phi}_{1,2}, \boldsymbol{\phi}_{2,2}, \dots, \boldsymbol{\phi}_{M_k,2}, \dots, \boldsymbol{\phi}_{M_k,L}]$, and $\boldsymbol{\psi}_{1:L} = [\boldsymbol{\psi}_1, \boldsymbol{\psi}_2, \dots, \boldsymbol{\psi}_L]$. The joint prior is,

$$p(\xi, \boldsymbol{\psi}_{1:L}, \boldsymbol{\phi}_{1:M_k,1:L}) = p(\xi) \prod_{k=1}^{L} \left\{ p(\boldsymbol{\psi}_k \mid \xi) \left[\prod_{j=1}^{M_k} p(\boldsymbol{\phi}_{j,k} \mid \boldsymbol{\psi}_k) \right] \right\}. \tag{2}$$

The likelihood is obtained through integrating out the individual cell parameters,

$$p(\mathfrak{D} \mid \xi, \boldsymbol{\psi}_{1:L}, \boldsymbol{\phi}_{1:M_k,1:L}) = \prod_{k=1}^{L} \prod_{j=1}^{M_k} \left\{ \int p(D_{j,k} \mid \boldsymbol{\theta}_{1:N_{j,k},j,k}) \left[\prod_{i=1}^{N_{j,k}} p(\boldsymbol{\theta}_{i,j,k} \mid \boldsymbol{\phi}_{j,k}) \, \mathrm{d}\boldsymbol{\theta}_{i,j,k} \right] \right\}. \tag{3}$$

Standard Bayesian tools have be applied to sample from the posterior ((1)) in the joint space $\boldsymbol{\Phi}^{M \times L} \times \boldsymbol{\Psi}^L \times \boldsymbol{\Xi}$. However, the correlation structures could be quite complex and the likelihood evaluation could be challenging due to the $\sum_{k=1}^{k=L} M_k$ integrals over $\boldsymbol{\Theta}^{N_{j,k}}$.
