# Supplementary material for: A multilevel hierarchical framework for quantification of experimental heterogeneity in population snapshot data
Source: PLoS Comput Biol. 2026 Jun 15;22(6):e1014379. doi: 10.1371/journal.pcbi.1014379 (PMC13286279; doi:10.1371/journal.pcbi.1014379)
Supplement: S2 Appendix — (PDF) [file pcbi.1014379.s002.pdf]

Supporting information for “A multilevel hierarchical framework for quantification of experimental heterogeneity”

David J. Warne[1*], Xiangrun Zhu[1], Thomas P. Steele[1], Stuart T. Johnston[2], Scott A. Sisson[3], Matthew Faria[4], Ryan J. Murphy[5], Alexander P. Browning[2,6]

1 School of Mathematical Sciences, Queensland University of Technology, Brisbane, Australia
2 School of Mathematics and Statistics, The University of Melbourne, Melbourne, Australia
3 School of Mathematics and Statistics, University of New South Wales, Sydney, Australia
4 Department of Biomedical Engineering, The University of Melbourne, Melbourne, Australia
5 School of Mathematical Sciences, Adelaide University, Adelaide, Australia
6 Mathematical Institute, University of Oxford, Oxford, United Kingdom

* david.warne@qut.edu.au

S2 Appendix. Approximate Bayesian Computation

The two level hierarchical model is presented in the main manuscript that captures heterogeneity both between replicates, characterised by $p(\cdot \mid \boldsymbol{\psi})$, and within the cell subpopulation of each replicate, characterised by $p_1(\cdot \mid \boldsymbol{\phi}_1), p_2(\cdot \mid \boldsymbol{\phi}_2), \ldots, p_M(\cdot \mid \boldsymbol{\phi}_M)$., we aim to infer the hyper-parameters, $\boldsymbol{\psi}, \boldsymbol{\phi}_1, \boldsymbol{\phi}_2, \ldots \boldsymbol{\phi}_M$. That is,

$$p(\boldsymbol{\psi}, \boldsymbol{\phi}_1, \ldots \boldsymbol{\phi}_M \mid \mathcal{D}) \propto p(\mathcal{D} \mid \boldsymbol{\psi}, \boldsymbol{\phi}_1, \ldots \boldsymbol{\phi}_M) p(\boldsymbol{\psi}, \boldsymbol{\phi}_1, \ldots \boldsymbol{\phi}_M), \tag{1}$$

where the joint prior that enforces the hierarchical structure of the model is given by

$$p(\boldsymbol{\psi}, \boldsymbol{\phi}_1, \ldots \boldsymbol{\phi}_M) = p(\boldsymbol{\psi}) \prod_{j=1}^{M} p(\boldsymbol{\phi}_j \mid \boldsymbol{\psi}), \tag{2}$$

and the likelihood is

$$p(\mathcal{D} \mid \boldsymbol{\psi}, \boldsymbol{\phi}_1, \ldots \boldsymbol{\phi}_M) = \prod_{j=1}^{M} \left\{ \int_{\boldsymbol{\Theta}^{N_j}} p(D_j \mid \Theta_j) p_j(\Theta_j \mid \boldsymbol{\phi}_j) \left[\prod_{i=1}^{N_j} \mathrm{d}\boldsymbol{\theta}_{i,j} \right] \right\}, \tag{3}$$

with $p_j(\Theta_j \mid \boldsymbol{\phi}_j) = \prod_{i=1}^{N_j} p_j(\boldsymbol{\theta}_{i,j} \mid \boldsymbol{\phi}_j)$.

In Eq. (3) we integrate out the individual cell parameters (the $\boldsymbol{\theta}_{i,j}$'s) within the likelihood function. This leads to a complex likelihood evaluation while reducing the dimension of the inference problem substantially to the space of hyper-parameters $\boldsymbol{\Phi}^M \times \boldsymbol{\Psi}$. An alternative and equivalent formulation is to perform inference over the entire parameter space then perform the marginalisation over the full posterior, yielding

$$p(\boldsymbol{\psi}, \boldsymbol{\phi}_1, \ldots \boldsymbol{\phi}_M \mid \mathcal{D}) = \int_{\boldsymbol{\Theta}^{\sum_{j=1}^{M} N_j}} p(\boldsymbol{\psi}, \boldsymbol{\phi}_1, \ldots \boldsymbol{\phi}_M, \Theta_1, \ldots, \Theta_M \mid \mathcal{D}) \left[\prod_{j=1}^{M} \prod_{i=1}^{N_j} \mathrm{d}\boldsymbol{\theta}_{i,j} \right] \tag{4}$$

with

$$p(\boldsymbol{\psi}, \boldsymbol{\phi}_1, \dots \boldsymbol{\phi}_M, \Theta_1, \dots, \Theta_M \mid \mathcal{D}) \propto p(\mathcal{D} \mid \boldsymbol{\psi}, \boldsymbol{\phi}_1, \dots \boldsymbol{\phi}_M, \Theta_1, \dots, \Theta_M) \times p(\boldsymbol{\psi}, \boldsymbol{\phi}_1, \dots \boldsymbol{\phi}_M, \Theta_1, \dots, \Theta_M). \tag{5}$$

Here, we have the joint prior and likelihood,

$$p(\boldsymbol{\psi}, \boldsymbol{\phi}_1, \dots \boldsymbol{\phi}_M, \Theta_1, \dots, \Theta_M) = p(\boldsymbol{\psi}) \prod_{j=1}^{M} p_j(\Theta_j \mid \boldsymbol{\phi}_j) p(\boldsymbol{\phi}_j \mid \boldsymbol{\psi}), \tag{6}$$

$$p(\mathcal{D} \mid \boldsymbol{\psi}, \boldsymbol{\phi}_1, \dots \boldsymbol{\phi}_M, \Theta_1, \dots, \Theta_M) = \prod_{j=1}^{M} p(D_j \mid \Theta_j). \tag{7}$$

In this formulation, the likelihood function (Eq. (7)) is relatively simple to evaluate (up to the complexity in obtaining a solution to the ODE system in the main manuscript. However, it requires posterior sampling over the full space $\boldsymbol{\Theta}^{\sum_{j=1}^{M} N_j} \times \boldsymbol{\Phi}^M \times \boldsymbol{\Psi}$, which is challenging for standard Monte Carlo methods.

To maintain the advantages of both formulations, that is, the lower dimensional inference problem from Equations (1)–(3) and the direct likelihood calculation from (4–(7)), we adopt an Approximate Bayesian computation (ABC) approach [1–3]. That is, we approximate Eq. (1) using the ABC posterior,

$$p_\epsilon(\boldsymbol{\psi}, \boldsymbol{\phi}_1, \dots, \boldsymbol{\phi}_M \mid \mathcal{D}) \propto \mathbb{P}(\rho(\mathcal{D}, \mathcal{D}_s) \leq \epsilon \mid \boldsymbol{\phi}_1, \dots, \boldsymbol{\phi}_M) p(\boldsymbol{\phi}_1, \dots, \boldsymbol{\phi}_M \mid \boldsymbol{\psi}) p(\boldsymbol{\psi}). \tag{8}$$

where $\mathcal{D}_s$ is simulated data and $\rho(\mathcal{D}, \mathcal{D}_s)$ is a distribution matching discrepancy metric based on the Anderson-Darling distance,

$$\rho(\mathcal{D}, \mathcal{D}_s) = \frac{1}{M} \sum_{j=1}^{M} \sum_{k=1}^{n} A(\mathcal{Y}_j(t_k), \mathcal{Y}_j^s(t_k)), \tag{9}$$

where $\mathcal{Y}_j(t_k) = [Y_{1,j}(t_k), Y_{2,j}(t_k), \dots, Y_{N_j,j}(t_k)]$ is the real data snapshot for replicate j at time t_k, and $\mathcal{Y}_j^s(t_k) = [Y_{1,j}^s(t_k), Y_{2,j}^s(t_k), \dots, Y_{N_j,j}^s(t_k)]$ is the simulated data snapshot for replicate j at time t_k. Here, the function $A(\mathcal{X}, \mathcal{Y})$ is the Anderson-Darling distance,

$$A(\mathcal{X}, \mathcal{Y}) = \left[-N - \sum_{i=1}^{N} \frac{2i-1}{N} \log\left(\hat{F}_{\mathcal{X}}\left(Y_i\right)\right) + \log\left(1 - \hat{F}_{\mathcal{X}}\left(Y_{N+1-i,j}\right)\right) \right]^{1/2}, \tag{10}$$

where $\mathcal{X} = \{X_1, X_2, \dots_N\}$ and $\mathcal{Y} = \{Y_1, Y_2, \dots, Y_N\}$ are two sample sets and $\hat{F}_{\mathcal{X}}(x)$ is the empirical cumulative distribution for $\mathcal{X}$, that is
$\hat{F}_{\mathcal{X}}(x) = \mathbb{P}(X \leq x) = \frac{1}{N} \sum_{i=1}^{N} \mathbb{1}_{(-\infty, x]}(X_i)$.

We have that $p_\epsilon(\boldsymbol{\phi}_1, \boldsymbol{\phi}_2, \dots, \boldsymbol{\phi}_M, \psi \mid \mathcal{D}) \to p(\boldsymbol{\psi}, \boldsymbol{\phi}_1, \boldsymbol{\phi}_2, \dots \boldsymbol{\phi}_M \mid \mathcal{D})$ as $\epsilon \to 0$. The approach is intuitively encapsulated in the ABC rejection sampling scheme shown in Alg. 1 for clarity. However, in practice it is not feasible to implement this method directly for small ϵ which is required for accuracy. Primarily this is due to the accepance rate the scales with $\mathcal{O}(\epsilon^d)$ where d is the dimension of the data space, that is, $d \approx \sum_{j=1}^{M} N_j$.

Algorithm 1 ABC rejection sampling for hierarchical flow cytometry analysis.

Draws $\mathcal{M}$ i.i.d. samples from the ABC approximate posterior $p_\epsilon(\boldsymbol{\psi}, \boldsymbol{\phi}_1, \boldsymbol{\phi}_2, \ldots, \boldsymbol{\phi}_M \mid \mathcal{D})$ (Eq. (8)) given parametric distributions for the joint prior, the dynamic model and observation process.

for $k \in [1, 2 \ldots, \mathcal{M}]$ **do**
    repeat
        Sample global hyper parameters $\boldsymbol{\psi}^* \sim p(\cdot)$;
        for $j \in [1, 2, \ldots, M]$ **do**
            Sample group hyper parameters $\boldsymbol{\phi}_j^* \sim p(\cdot \mid \boldsymbol{\psi}^*)$;
            Sample population parameters $\Theta_j^* \sim p_j(\cdot \mid \boldsymbol{\phi}_j^*)$;
            Given Θ_j^* Solve model ODEs for $\mathcal{X}_j^*(t_1), \mathcal{X}_j^*(t_2), \ldots, \mathcal{X}_j^*(t_n)$;
            Sample observation processes to obtain $D_j^* = [\mathcal{Y}_j^*(t_1), \mathcal{Y}_j^*(t_2), \ldots, \mathcal{Y}_j^*(t_n)]$;
        end for
        $\mathcal{D}_s \leftarrow [D_1, D_2, \ldots, D_M]$;
    until $\rho(\mathcal{D}, \mathcal{D}_s) < \epsilon$
    $\boldsymbol{\psi}^k \leftarrow \boldsymbol{\psi}^*$ and $\boldsymbol{\phi}_j^k \leftarrow \boldsymbol{\phi}_j^*$ for $j = 1, 2, \ldots, M$;
end for
return $(\boldsymbol{\psi}^k, \boldsymbol{\phi}_1^k, \boldsymbol{\phi}_2^k, \ldots, \boldsymbol{\phi}_M^k)$ for $k = 1, 2, \ldots, \mathcal{M}$.

Due to the infeasibility of Alg. 1 for our application, we implement an adaptive ABC scheme based on sequential Monte Carlo (SMC) methods of Drovandi and Pettitt [4] that refines ϵ through sequential importance resampling. The algorithm initially generates $\mathcal{M}_p$ samples, referred to as particles, from the prior and then applies sequential importance resampling to propagate particles through a sequences of T ABC posterior distributions such that $\epsilon_t > \epsilon_{t+1}$ for all $t \in 0, 1, \ldots, T-1$. The specific sequence of thresholds is determined adaptively so that exactly $\mathcal{M}_a = a\mathcal{M}_p$ particles are accepted at each step, where $a \in (0, 1]$ such that $\mathcal{M}_a \in \mathbb{Z}^+$. After resampling, particles are mutated based on an Markov chain Monte Carlo (MCMC) kernel that is adapted according a standard optimal scaling rule [5]. The number of MCMC steps, R, performed at SMC iteration t is chosen such that the probability of the chain moving accepting at least one proposal is $1 - c$ where $c \in (0, 1)$. This requires an estimate of the acceptance probability which is obtained for a small number of trials, R_{trial}, across all particles. For our implementation, the tuning parameters were set to $a = 0.5$, $c = 0.01$, and $R_{\text{trial}} = 50$ and $\mathcal{M}_p = 2{,}000$. Alg. 2 provides an implementation of the ABC SMC approach.

Algorithm 2 Adaptive SMC sampler for approximate Bayesian computation

Initialise $\mathcal{M}_a = a\mathcal{M}_p$, $\mathcal{M}_\ell = \mathcal{M}_p - \mathcal{M}_a$
for $k \in [1, 2, \ldots, \mathcal{M}_p]$ **do**
  Sample prior, $\boldsymbol{\psi}^k \sim p(\cdot)$, and $\boldsymbol{\phi}_1^k, \ldots, \boldsymbol{\phi}_M^k \sim p(\cdot \mid \boldsymbol{\psi}^k)$;
  Simulate data, $\mathcal{D}_s \sim s(\cdot \mid \boldsymbol{\psi}^k, \boldsymbol{\phi}_1^k, \ldots, \boldsymbol{\phi}_M^k)$;
  Set $\rho_k \leftarrow \rho(\mathcal{D}, \mathcal{D}_s)$;
end for
repeat
  Sort particles $\{(\boldsymbol{\psi}^k, \boldsymbol{\phi}_1^k, \ldots, \boldsymbol{\phi}_M^k, \rho_k)\}_{k=1}^{\mathcal{M}_p}$, such that $\rho_k \leq \rho_{k+1}$ for all k;
  Remove particles $\{(\boldsymbol{\psi}^k, \boldsymbol{\phi}_1^k, \ldots, \boldsymbol{\phi}_M^k, \rho_k)\}_{k=\mathcal{M}_\ell+1}^{\mathcal{M}_p}$ an set $\epsilon \leftarrow \rho_{\mathcal{M}_\ell}$;
  Resample particles $\{(\boldsymbol{\psi}^k, \boldsymbol{\phi}_1^k, \ldots, \boldsymbol{\phi}_M^k)\}_{k=\mathcal{M}_\ell+1}^{\mathcal{M}_p}$ from $\{(\boldsymbol{\psi}^k, \boldsymbol{\phi}_1^k, \ldots, \boldsymbol{\phi}_M^k)\}_{j=1}^{\mathcal{M}_\ell}$ with replacement;
  Estimate sample covariance, $\hat{\boldsymbol{\Sigma}}$, of particles $\{(\boldsymbol{\psi}^k, \boldsymbol{\phi}_1^k, \ldots, \boldsymbol{\phi}_M^k)\}_{k=1}^{\mathcal{M}_p}$.
  Adapt proposal kernel $q(\mathbf{u} \mid \mathbf{v}) = \phi\left(\mathbf{u}; \mathbf{v}, \dfrac{2.38^2}{d_{\boldsymbol{\Psi}} + M d_{\boldsymbol{\Phi}}} \hat{\boldsymbol{\Sigma}}\right)$, where $\phi(\cdot; \boldsymbol{\mu}, \boldsymbol{\Sigma})$ is a multivariate Gaussian density function, $d_{\boldsymbol{\Psi}}$ is the dimension of $\boldsymbol{\psi} \in \boldsymbol{\Psi}$ and $d_{\boldsymbol{\Psi}}$ is the dimension of $\boldsymbol{\phi} \in \boldsymbol{\Phi}$;
  Set $p_{\text{acc}} \leftarrow 0$;
  for $k \in [\mathcal{M}_\ell + 1, \mathcal{M}_\ell + 2, \ldots, \mathcal{M}_p]$ **do**
    for $r \in [1, 2, \ldots, R_{\text{trial}}]$ **do**
      Generate proposal, $(\boldsymbol{\psi}^*, \boldsymbol{\phi}_1^*, \ldots, \boldsymbol{\phi}_M^*) \sim q(\cdot \mid \boldsymbol{\psi}^k, \boldsymbol{\phi}_1^k, \ldots, \boldsymbol{\phi}_M^k)$;
      $\alpha \leftarrow \min\left(1, \dfrac{p(\boldsymbol{\psi}^*, \boldsymbol{\phi}_1^*, \ldots, \boldsymbol{\phi}_M^*) q(\boldsymbol{\psi}^k, \boldsymbol{\phi}_1^k, \ldots, \boldsymbol{\phi}_M^k \mid \boldsymbol{\psi}^*, \boldsymbol{\phi}_1^*, \ldots, \boldsymbol{\phi}_M^*)}{p(\boldsymbol{\psi}^k, \boldsymbol{\phi}_1^k, \ldots, \boldsymbol{\phi}_M^k) q(\boldsymbol{\psi}^*, \boldsymbol{\phi}_1^*, \ldots, \boldsymbol{\phi}_M^* \mid \boldsymbol{\psi}^k, \boldsymbol{\phi}_1^k, \ldots, \boldsymbol{\phi}_M^k)}\right)$;
      Sample $u \sim \mathcal{U}(0, 1)$;
      if $u \leq \alpha$ **then**
        Simulate model $\mathcal{D}_s \sim s(\cdot \mid \boldsymbol{\psi}^*, \boldsymbol{\phi}_1^*, \ldots, \boldsymbol{\phi}_M^*)$;
        if $\rho(\mathcal{D}, \mathcal{D}_s) \leq \epsilon$ **then**
          Set $(\boldsymbol{\psi}^k, \boldsymbol{\phi}_1^k, \ldots, \boldsymbol{\phi}_M^k) \leftarrow (\boldsymbol{\psi}^*, \boldsymbol{\phi}_1^*, \ldots, \boldsymbol{\phi}_M^*)$,$\rho_k \leftarrow \rho(\mathcal{D}, \mathcal{D}_s)$;
          Update $p_{\text{acc}} \leftarrow p_{\text{acc}} + (R_{trial}\mathcal{M}_a)^{-1}$;
        end if
      end if
    end for
  end for
  Set $R \leftarrow \log c / \log(1 - p_{\text{acc}})$;
  for $k \in [\mathcal{M}_\ell + 1, \mathcal{M}_\ell + 2, \ldots, \mathcal{M}_p]$ **do**
    for $r \in [1, 2, \ldots, R_{\text{trial}}]$ **do**
      Generate proposal, $(\boldsymbol{\psi}^*, \boldsymbol{\phi}_1^*, \ldots, \boldsymbol{\phi}_M^*) \sim q(\cdot \mid \boldsymbol{\psi}^k, \boldsymbol{\phi}_1^k, \ldots, \boldsymbol{\phi}_M^k)$;
      $\alpha \leftarrow \min\left(1, \dfrac{p(\boldsymbol{\psi}^*, \boldsymbol{\phi}_1^*, \ldots, \boldsymbol{\phi}_M^*) q(\boldsymbol{\psi}^k, \boldsymbol{\phi}_1^k, \ldots, \boldsymbol{\phi}_M^k \mid \boldsymbol{\psi}^*, \boldsymbol{\phi}_1^*, \ldots, \boldsymbol{\phi}_M^*)}{p(\boldsymbol{\psi}^k, \boldsymbol{\phi}_1^k, \ldots, \boldsymbol{\phi}_M^k) q(\boldsymbol{\psi}^*, \boldsymbol{\phi}_1^*, \ldots, \boldsymbol{\phi}_M^* \mid \boldsymbol{\psi}^k, \boldsymbol{\phi}_1^k, \ldots, \boldsymbol{\phi}_M^k)}\right)$;
      Sample $u \sim \mathcal{U}(0, 1)$;
      if $u \leq \alpha$ **then**
        Simulate model $\mathcal{D}_s \sim s(\cdot \mid \boldsymbol{\psi}^*, \boldsymbol{\phi}_1^*, \ldots, \boldsymbol{\phi}_M^*)$;
        if $\rho(\mathcal{D}, \mathcal{D}_s) \leq \epsilon$ **then**
          Set $(\boldsymbol{\psi}^k, \boldsymbol{\phi}_1^k, \ldots, \boldsymbol{\phi}_M^k) \leftarrow (\boldsymbol{\psi}^*, \boldsymbol{\phi}_1^*, \ldots, \boldsymbol{\phi}_M^*)$,$\rho_k \leftarrow \rho(\mathcal{D}, \mathcal{D}_s)$;
          Update $p_{\text{acc}} \leftarrow p_{\text{acc}} + (R_{trial}\mathcal{M}_a)^{-1}$;
        end if
      end if
    end for
  end for
until $p_{\text{acc}} < p_{\min}$

References

1. Sisson SA, Fan Y, Beaumont M. Approximate Bayesian Computation. Taylor Francis Group; 2018.

2. Sunnåker M, Busetto AG, Numminen E, Corander J, Foll M, Dessimoz C. Approximate Bayesian Computation. PLoS Computational Biology. 2013 Jan;9(1):e1002803. doi:10.1371/journal.pcbi.1002803.

3. Warne DJ, Baker RE, Simpson MJ. Simulation and inference algorithms for stochastic biochemical reaction networks: from basic concepts to state-of-the-art. Journal of The Royal Society Interface. 2019 Feb;16(151):20180943. doi:10.1098/rsif.2018.0943.

4. Drovandi CC, Pettitt AN. Estimation of Parameters for Macroparasite Population Evolution Using Approximate Bayesian Computation. Biometrics. 2010 Mar;67(1):225-33. doi:10.1111/j.1541-0420.2010.01410.x.

5. Roberts GO, Rosenthal JS. Optimal scaling for various Metropolis-Hastings algorithms. Statistical Science. 2001 Nov;16(4). doi:10.1214/ss/1015346320.
